# Physiological responses of *Daphnia pulex *to acid stress

**DOI:** 10.1186/1472-6793-9-9

**Published:** 2009-04-21

**Authors:** Anna K Weber, Ralph Pirow

**Affiliations:** 1 Institute of Zoophysiology, University of Münster, Münster, Germany

## Abstract

**Background:**

Acidity exerts a determining influence on the composition and diversity of freshwater faunas. While the physiological implications of freshwater acidification have been intensively studied in teleost fish and crayfish, much less is known about the acid-stress physiology of ecologically important groups such as cladoceran zooplankton. This study analyzed the extracellular acid-base state and CO_2 _partial pressure (*P*_CO2_), circulation and ventilation, as well as the respiration rate of *Daphnia pulex *acclimated to acidic (pH 5.5 and 6.0) and circumneutral (pH 7.8) conditions.

**Results:**

*D. pulex *had a remarkably high extracellular pH of 8.33 and extracellular *P*_CO2 _of 0.56 kPa under normal ambient conditions (pH 7.8 and normocapnia). The hemolymph had a high bicarbonate concentration of 20.9 mM and a total buffer value of 51.5 meq L^-1 ^pH^-1^. Bicarbonate covered 93% of the total buffer value. Acidic conditions induced a slight acidosis (ΔpH = 0.16–0.23), a 30–65% bicarbonate loss, and elevated systemic activities (tachycardia, hyperventilation, hypermetabolism). pH 6.0 animals partly compensated the bicarbonate loss by increasing the non-bicarbonate buffer value from 2.0 to 5.1 meq L^-1 ^pH^-1^. The extracellular *P*_CO2 _of pH 5.5 animals was significantly reduced to 0.33 kPa, and these animals showed the highest tolerance to a short-term exposure to severe acid stress.

**Conclusion:**

Chronic exposure to acidic conditions had a pervasive impact on *Daphnia's *physiology including acid-base balance, extracellular *P*_CO2_, circulation and ventilation, and energy metabolism. Compensatory changes in extracellular non-bicarbonate buffering capacity and the improved tolerance to severe acid stress indicated the activation of defense mechanisms which may result from gene-expression mediated adjustments in hemolymph buffer proteins and in epithelial properties. Mechanistic analyses of the interdependence between extracellular acid-base balance and CO_2 _transport raised the question of whether a carbonic anhydrase (CA) is involved in the catalysis of the  reaction, which led to the discovery of 31 CA-genes in the genome of *D. pulex*.

## Background

Freshwater acidification is an important stressor that affects the structure of zooplankton communities in lake ecosystems. Acidification may arise from natural processes such as spring acid episodes [1,2], acid rock drainage [3] and volcanism [4,5], or from anthropogenic activities including fossil fuels burning [6,7], agriculture measures [8], dredging of waterways [9,10] and mining-related processes (e.g. acid mine drainage, recultivation and flooding of former mining pits) [11,12]. pH levels below 5–6 generally decrease the zooplankton species richness compared to circumneutral pH conditions [13-16]. Among the zooplankton, crustaceans of the genus *Daphnia *are usually less abundant in acidified lakes while other (non-daphnid) cladocerans, calanoid copepods, and insects (e.g. *Chaoborus *larvae and corixids) may increase in importance or even become dominating [15,17]. The mechanisms behind these changes in the zooplankton community structure are manifold. They include a differential physiological sensitivity to acid stress [14,18,19], a differential tolerance against toxic metal species [20-22], which become more soluble under acidic conditions, as well as altered biotic interactions arising from the effect of pH on phytoplankton and planktivore communities [14,23].

It is well-known that the physiological sensitivity of aquatic animals to acidic conditions is associated with iono- and osmoregulatory processes [24,25]. Faced with the continuous diffusive gain of water and loss of ions, freshwater animals generally have to minimize their whole-body permeability to water and/or ions and additionally require compensatory uptake mechanisms for sodium and chloride to maintain a steady-state ion balance [26]. While data on whole-body water permeability of freshwater zooplankton are essentially lacking [27-29], there is some information on sodium permeability. In general, acid-tolerant species such as aquatic insects have a relatively low sodium permeability in comparison to cladocerans [18,30-32]. In the acid-sensitive daphnids, the inability to survive long term below pH 5 is correlated with the net loss of body sodium due to an accelerated rate of Na^+ ^loss and a reduced rate of Na^+ ^uptake [33,34], a process that is additionally influenced by the ambient calcium concentration [35].

The acidification-induced impairment of sodium uptake in daphnids suggests that the transport of sodium across the epipodites – the *so-called *'branchial sacs' [36] – is linked with proton extrusion [33,35], as it is in the gills of other freshwater animals such as fish and crayfish [37-40]. The protons arise from the catalyzed hydration of CO_2 _by a cytoplasmic carbonic anhydrase in the ionoregulatory epithelia. This reaction produces  which is then excreted in exchange for chloride [41]. The interdependence between ionoregulatory processes, acid-base balance, and CO_2 _transport explains the strong impact of acid stress on the physiology of many freshwater animals. However, in contrast to the detailed information on teleost fish [24] and crayfish [25], the physiological implications of acid stress in daphnids have remained largely unexplored. Daphnids are important model organisms in ecotoxicology, and there is a growing interest in establishing mechanistic links between molecular stress responses and organismal stress responses [42-47]. Understanding the specific physiology of *Daphnia *may help to elucidate the modes of action of environmental toxicants [48,49].

The present study provides the experimental, methodical, and conceptual framework to analyze the acid-stress physiology of daphnids. Preliminary tests with *Daphnia pulex *yielded the appropriate acclimation conditions which guaranteed the survival, growth, and reproduction under acidic (pH 5.5 and 6.0) and circumneutral conditions (pH 7.8). Based on these stable laboratory populations, we determined the buffer characteristics from microliter hemolymph samples, analyzed the extracellular acid-base state by microspectralfluorometry using the pH-sensitive dye cSNARF-1, and studied the responses to a short-term exposure to severe acid stress (pH 3–4). Circulation, ventilation and respiration were additionally analyzed and served as diagnostic indicators for the interpretation of acid-base disturbances. Moreover, reproduction was monitored to assess acidification-induced changes in maintenance costs and energy-and-mass budget. Finally, the implications of the presence or absence of an extracellular carbonic anhydrase for acid-base balance and circulatory CO_2 _transport are discussed.

## Results and discussion

### Acid-base balance under normal conditions

**Figure 1:**
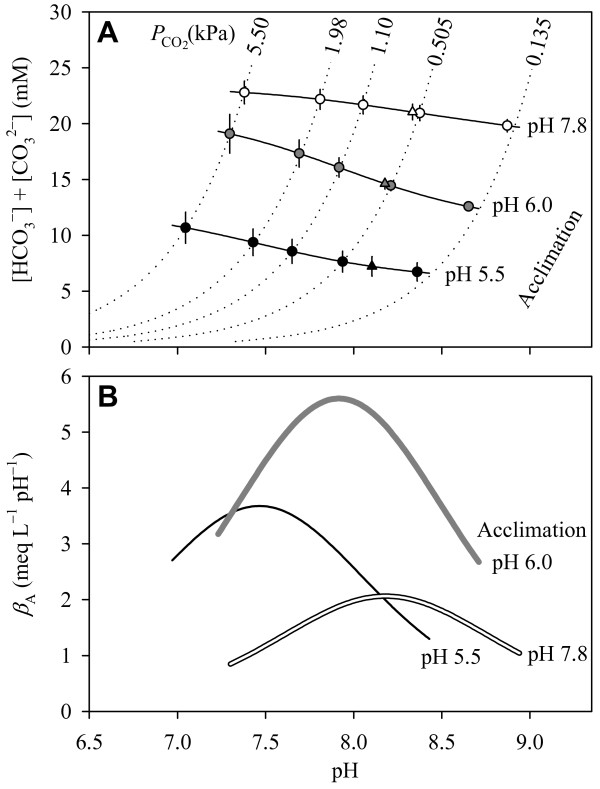
**Hemolymph buffer curves and non-bicarbonate buffer values**. (A) Hemolymph buffer curves of animals raised at 20°C at pH 7.8 (open symbols), pH 6.0 (grey-filled symbols), and pH 5.5 (filled symbols). The symbols represent the mean of a three-fold determination. The error in the concentration of chemically bound CO_2 _arises from the standard error in the calculation of CO_2 _partial pressure (*P*_CO2_) for a given pH (Table 1). The solid lines were calculated using the equations 1–3 and the means of the individual (curve-specific) parameter values given in Table 1. Dotted lines represent the *P*_CO2 _isobars. The triangles indicate the *in vivo *acid-base state of the respective acclimation groups. (B) Non-bicarbonate buffer values (*β*_A_) calculated from equation 4.

A rather alkaline extracellular pH of 8.334 ± 0.006 (mean ± S.E., *N *= 4) was measured in the heart region of animals which were raised and examined under normal conditions (i.e. ambient pH = 7.8, *P*_CO2 _= 0.035 kPa, and 20°C). By taking the characteristics and the variability of the hemolymph buffer curves (Table 1) into account, the mean *in vivo *pH corresponded to an equilibrium *P*_CO2 _of 0.56 ± 0.02 kPa (means ± S.E., *N *= 3 buffer curves) and a hemolymph bicarbonate concentration of 20.9 ± 0.7 mM (Table 2 and Figure 1A, open triangle). The derived *P*_CO2 _value is a representative measure of the extracellular *P*_CO2 _in the heart reagion as long as the CO_2_+H_2_O↔H^+^+ reaction in the hemolymph can reasonably be assumed to be in equilibrium. The information on the hemolymph buffer curves and the extracellular pH was used to assess the capacity of the extracellular compartment to buffer hydrogen ions of metabolic origin. The hemolymph had a total buffer value (*β*_T_) of 51.5 meq L^-1 ^pH^-1 ^(Table 2). Bicarbonate covered 93% of *β*_T_, and the non-bicarbonate buffer value (*β*_A_) was 2.0 meq L^-1 ^pH^-1^.

**Table 1:** Analysis of hemolymph buffer curves and determination of *P*_CO2 _from pH.

**Group**	**SID** **(meq L^-1^)**	***C*_A _(mM)**	**p***K'*_A_	**rmse**
pH 7.8 acclimation	21.1 ± 0.2	2.3 ± 0.3	8.18 ± 0.11	0.003
	24.9 ± 0.3	5.2 ± 0.3		
	23.9 ± 0.2	3.2 ± 0.3		

pH 6.0 acclimation	20.9 ± 0.8	10.4 ± 0.9	7.92 ± 0.11	0.011
	25.0 ± 1.2	14.4 ± 1.1		
	17.1 ± 0.5	4.4 ± 0.7		

pH 5.5 acclimation	13.6 ± 0.4	7.4 ± 0.4	7.46 ± 0.06	0.006
	9.2 ± 0.2	4.4 ± 0.2		
	14.6 ± 0.4	7.4 ± 0.4		

Buffer curves were obtained by measuring the pH of hemolymph samples in dependence on CO_2 _partial pressure (*P*_CO2_). The three buffer curves of each acclimation group were simultaneously fitted by the binary buffer model (equation 1), using p*K'*_A _as a shared parameter. This means that the value of this parameter was forced to be the same for all three buffer curves. Given are the best-fit parameter values (mean ± standard error) for the strong ion difference (SID), the concentration (*C*_A_) and p*K'*_A _value of the non-bicarbonate buffer, and the the standard error of the fit (rmse, root mean squared error). The number for the degrees of freedom (i.e. the number of data points minus the number of fitted parameters) was 8. The reverse determination of *P*_CO2 _from pH is exemplified as follows. Given the *in vivo *pH of 8.334, the three calibration buffer curves of the pH 7.8 acclimation group yielded *P*_CO2 _values of 0.519 ± 0.005 kPa, 0.573 ± 0.006 kPa, and 0.575 ± 0.005 kPa. These individual means were finally averaged to yield an overall mean of 0.556 kPa with a standard error of 0.018 kPa. This standard error therefore reflects the variability among the buffer curves.

**Table 2:** Comparison of acid-base, systemic and respiratory variables among the different acclimation groups.

**Physiological Variable**	**pH 7.8 acclimation**	**pH 6.0 acclimation**	**pH 5.5 acclimation**	**Significant difference among acclimation groups**
pH	8.334 ± 0.006^†^	8.177 ± 0.025	8.104 ± 0.008^#^	***

(kPa)	0.56 ± 0.02	0.56 ± 0.02	0.33 ± 0.04^#^	***

(mM)	20.9 ± 0.7	14.6 ± 0.5^#^	7.2 ± 0.9^#^	***

(mM)	0.153 ± 0.005	0.074 ± 0.003	0.031 ± 0.007	***

*β*_A _(meq L^-1 ^pH^-1^)	2.0	5.1	2.2	not tested

*β*_B _(meq L^-1 ^pH^-1^)	48.1	33.5	16.6	not tested

*β*_C _(meq L^-1 ^pH^-1^)	0.70	0.34	0.14	not tested

*β*_T _(meq L^-1 ^pH^-1^)	51.5	39.4	19.1	not tested

*f*_H _(min^-1^)	205 ± 10^†^	246 ± 18	299 ± 12^#^	**

*f*_A _(min^-1^)	374 ± 32^†^	427 ± 58	500 ± 6^#^	*

(nmol h^-1 ^mm^-3^)	1.53 ± 0.09	1.95 ± 0.07^†#^	1.41 ± 0.14^†^	**

*P*_CO2_, CO_2 _partial pressure; *β*_A_, buffer value of the non-bicarbonate buffer; *β*_B _and *β*_C_, bicarbonate and carbonate buffer value; *β*_T_, total buffer value (= *β*_A _+ *β*_B _+ 2*β*_C_); *f*_H_, heart rate; *f*_A_, appendage beating rate; , specific oxygen consumption rate (per cubic body length). Data are expressed as means ± standard error, except for the buffer values which are given as means. The number of independent determinations (*N*) is 3, if not otherwise indicated. The *P*_CO2 _was calculated from the mean pH value and the three corresponding buffer curves (Table 1). Asterisks indicate significant differences among the acclimation groups (*P < 0.05, **P < 0.01, ***P < 0.001). ^#^significant difference between an acid-stress (pH 6.0 or pH 5.5) group and the control (pH 7.8) group. ^†^*N *= 4.

Given the extracellular pH of 8.334, which is markedly higher than the circumneutral values of other water-breathing crustaceans (Table 3) [50-70], one is tempted to assume that *Daphnia pulex *is in a state of permanent respiratory alkalosis. Indeed, the filter-feeding mode of life of daphnids is inevitably associated with high ventilation rates (e.g. 0.75 mm^3 ^s^-1 ^[mm^-3 ^body volume] for *D. magna*) [71], which should favor the wash-out of carbon dioxide from the hemolymph. However, the present study gave no indication for a respiratory hypocapnia in *D. pulex*, since the extracellular pH suggested an equilibrium *P*_CO2 _of 0.56 kPa, which is higher than the typical *P*_CO2 _values (0.2–0.5 kPa, Table 3) in the prebranchial and postbranchial hemolymph of other water-breathing crustaceans. If the equilibrium *P*_CO2 _in the postbranchial hemolymph of *D. pulex *would approach the low value of, say, 0.2 kPa, an extreme alkalosis (pH 8.75) would occur. Taking the scaling relationship between metabolic rate and body size into account [72], the exceptional acid-base state of these small crustaceans seems to be determined by two main factors: (i) a high, specific metabolic rate, which contributes to the elevated *P*_CO2 _levels, and (ii) a high bicarbonate buffer value, which might be a pre-adaptive feature to cope with a highly variable, physiologically challenging environment.

**Table 3:** Acid-base status in Crustacea.

**Group/Species**	**pH**	[] **(mM)**	***P*_CO2 _(kPa)**	**arterial/** **venous**	***β*_A _(mM pH^-1^)**	***T *(°C)**	**Medium**	**Mode of ** **Life**	**Reference**
**Branchiopoda**									

*Daphnia pulex*	8.33	21.0	0.56		1.75	20	FW	A	§

*Daphnia magna*	8.44	13.4	0.28		0.5	20	FW	A	$

*Triops cancriformis*	7.52	7.6	1.36		2.1	20	FW	A	$

**Decapoda**									

*Astacus astacus*	7.78	5.2	0.27	a	6.3	15	FW	A	[79]

*Astacus leptodactylus*	7.87	4.5	0.26	v	11.6	13	FW	A	[61]

*Pacifastacus leniusculus*	7.95	8.8^#^	0.37	a	11.6	15	FW	A	[69]

*Austropotamobius pallipes*	7.90	6.9^#^	0.40	a	13.5	15	FW	A	[64]

*Orconectes rusticus*	7.87	5.8^#^	0.45	a		15	FW	A	[70]

*Orconectes propinquus*	7.75	7.0	0.37	a	8	10	FW	A	[78]

*Procambarus clarki*	7.93	9.9	0.49	a		15	FW	A	[77]

*Procambarus clarki*	8.17	17.8	0.44	a		15	FW	A	[77]

*Procambarus clarki*	7.75	7.0	0.40	a			FW	A	[25]

*Homarus vulgaris*	7.80	5.6	0.26	a	8	15	SW	A	[57]

*Homarus gammarus*	7.78	9.3	0.44	a	15	15	SW	A	[65]

*Palaemon elegans*	7.89	5.4^#^	0.17	a	16	15	SW	A	[59]

*Palaemon adspersus*	7.85	4–7	0.25	a	4–9	15	SW	A	[68]

*Penaeus japonicus*	7.58	6.0	0.44	a		18	SW	A	[52]

*Carcinus maenas*	7.82	3.9	0.15	v	13.3	15	SW	X	[66]

*Callinectes sapidus*	7.96	8.8	0.40	v	5	22	FW	A	[50]

*Scylla serrata*	7.68	7.5^#^	0.48	a	13.2	25	SW	A	[67]

*Necora puber*	7.90	6.6	0.19	v		15	SW	A	[62]

*Cancer magister*	7.73	4.5	0.25	a		17	SW	A	[56]

*Cancer productus*	7.89	9.0	0.30	a		10	SW	A	[53]

*Gecarcinus lateralis*	7.37	5.9	0.86	a		25	SW	T	[63]

*Cardisoma carnifex*	7.64	10.3^#^	0.93	a		28	SW	T	[51]

*Cyclograpsus lavauxi*	7.92	10.9	0.31	a		10	SW	X	[55]

*Leptograpsus variegatus*	7.90	5.4^#^	0.24	a		20	SW	X	[58]

*Holthusiana transversa*	7.33	9.5	0.80	a		25	FW	X	[54]

**Amphipoda**									

*Gammarus pulex*	8.00	12.4	0.40	a	2.6	12	FW	A	[60]

*Gammarus fossarum*	8.00	10.4	0.33	a	3.0	12	FW	A	[60]

Hemolymph acid-base status of various crustaceans in freshwater (FW) and seawater (SW) under aerated conditions at temperature *T*. Postbranchial (a = 'arterial') hemolymph was drawn from the pericardial sinus, whereas prebranchial (v = 'venous') samples were taken from the infrabranchial sinus at the base of the walking leg. For each species, the typical mode of life (A = aquatic, T = terrestrial, X = amphibious) is indicated. ^#^total CO_2_, ^§^this study, ^$^unpublished data.

One may argue that the *in vivo *results are to some extent influenced by the experimental procedures, which required the microinjection of a pH-sensitive dye into the circulatory system of immobilized animals. Previous studies [71,73-75] have shown that the immobilization does not induce any noticable physiological disturbances, provided that the animals have the chance to acclimate to the experimental conditions for at least 30 min. Immobilized animals of *D. magna*, for example, exhibit the typical resting values in heart rate (*f*_H_) and appendage beating rate (*f*_A_) and respond in a predictable manner to changes in abiotic [73-75] and biotic factors [71]. The microinjection procecure, however, is known to induce a bradycardia in *D. magna *[76] and it did so in *D. pulex*. Our microinjected control animals (pH 7.8 acclimation) had a *f*_H _of 205 ± 10 min^-1 ^(*N *= 4), which was significantly lower than that of non-injected animals (310 ± 28 min^-1^, *N *= 5; *t*-test: *P *= 0.01). In contrast, there was no significant effect on *f*_A _(injected: 374 ± 32 min^-1^, non-injected: 352 ± 51 min^-1^; *P *= 0.8). The slower *f*_H _was very likely caused by the increase in hemolymph viscosity due to the injection of the dye-coupled 70-kDa dextrans. Given the 34% reduction in *f*_H_, one may suppose a pertubation in the hemolymph partial pressures of respiratory gases including the *P*_CO2_. Theoretical analyses in terms of the CO_2 _transport model, which is described below, revealed that the mean extracellular *P*_CO2 _would be 8% smaller in the absence of a bradycardia. An effect of this magnitude does not invalidate the findings about the exceptional acid-base state of *D. pulex*.

### Physiological and visible effects of chronic exposure to acidic conditions

Animals raised and tested under acidic conditions (ambient pH 6.0 and pH 5.5) had extracellular pH values of 8.177 ± 0.025 and 8.104 ± 0.008 (*N *= 3 each), respectively. These values were 0.16–0.23 pH units lower than that of the control (pH 7.8 acclimated) animals. The differences in extracellular pH among the acclimation groups were statistically significant (Table 2). The extracellular *P*_CO2 _(0.56 ± 0.02 kPa) of the pH 6.0 acclimated animals was virtually the same as that of the control group. In contrast, pH 5.5 animals had a significantly lower extracellular *P*_CO2 _of 0.33 ± 0.04 kPa (Table 2).

The slight acidosis in the extracellular fluid was associated with a significant (30–65%) reduction in hemolymph bicarbonate concentration to 14.6 ± 0.5 mM in pH 6.0 animals and 7.2 ± 0.9 mM in pH 5.5 animals (Table 2 and Figure 1A, gray and black triangles). Reductions of similar relative magnitude have been observed in freshwater crayfish [77-79]. This depletion in hemolymph bicarbonate, by the entry of acidic equivalents from the ambient medium (see below), caused a proportional reduction in the bicarbonate buffer value (*β*_B_). The pH 6.0 animals partly compensated the 30% reduction in *β*_B _by increasing the non-bicarbonate buffer value (*β*_A_) from 2.0 to 5.1 meq L^-1 ^pH^-1^, while pH 5.5 animals experienced a 65% loss in *β*_B _(Table 2). Although the compensatory increase in *β*_A _was almost negligible, the pH 5.5 animals still had significant reserves in *β*_A _which are available in the case of a progressive acidosis (Figure 1B).

Heart rate (*f*_H_), ventilation rate (*f*_A_), and oxygen consumption rate () were additionally monitored as diagnostic indicators for the mechanistic interpretation of acid-base disturbances. Compared to the control group, animals raised and tested under pH 6.0 showed a 20% higher *f*_H_, a 14% higher *f*_A _and a 38% higher  (Table 2), supposedly to meet the increased maintenance requirements for ion regulation. These systemic adjustments had no influence on extracellular *P*_CO2_. The acidosis of the pH 6.0 animals (ΔpH = -0.16 units; Table 2) was therefore of metabolic rather than of respiratory origin. In agreement with the convention in acid-stress physiology [80], the term 'metabolic acidosis' is used here irrespective of whether the protons originate endogenously in connection with lactic acid production or exogenously, by the influx of H^+ ^down the large medium-to-hemolymph H^+ ^gradient. The metabolic acidosis was very likely caused by an influx of acidic equivalents from the ambient medium, since the sustained circulation and ventilation argue against the possibility of an activation of anaerobic support mechanisms.

Compared to the control group, the pH 5.5 animals experienced a 0.23-unit decrease in extracellular pH (Table 2), which can be characterized as metabolic acidosis with respiratory compensation as indicated by the reduced extracellular *P*_CO2_. Since the oxygen consumption rate (and consequently the CO_2 _production rate) did not change significantly in comparison to the control animals (Table 2), the main reasons for the reduced extracellular *P*_CO2 _are the 34–46% increased ventilation and perfusion rates (Table 2) as well as an enhanced permeability of the integument for respiratory gases, probably due to a thinner carapace. The latter explanation is consistent with the observation of the softer carapaces, which occurred only in the pH 5.5 animals. As in acid-stressed freshwater crayfish [81-83], the softer (jelly-like) carapace of pH 5.5 animals may indicate a poor calcification resulting from exoskeletal CaCO_3 _erosion and reduced calcium and basic equivalent () uptake during the postmoult stage.

One may wonder why the acidification-induced increase in  was only present in the pH 6.0 animals but absent in the pH 5.5 animals. The  is expressed here as specific rate (nmol h^-1 ^mm^-3^), which is normalized to cubic body length rather than to body weight. It is common practice to estimate the body weight of daphnids from body length using scaling relationships (e.g. [84]). However, we discarded this estimation because of the uncertainty about the influence of acidification on the relationship between body length and body weight. In the pH 5.5 animals, a reduction in the amount of metabolically active biomass (per cubic body length) could have masked the supposed extra costs for ion regulation. Evidence for this explanation comes from the comparison of brood sizes of those animals which were analyzed in the respiration experiment. The egg numbers of pH 5.5 animals (1.2 ± 0.6, range: 0–6, *N *= 12) were significantly lower than those of the pH 6.0 animals (9.1 ± 0.6, *N *= 12) and pH 7.8 animals (7.8 ± 1.0, *N *= 9) (Kruskal-Wallis test, *P *< 0.001). All eggs were of early developmental stage and accounted for very little respiration in the brooding females [85]. Nevertheless, the reduced allocation of resources into reproduction implies an acidification-induced disturbance in the energy and mass budgets of the pH 5.5 animals. Indeed, these animals showed the highest degree of transparency owing to the decreased appearance of orange-colored fat cells. Fat cells store carbohydrates and lipids [86-88], the latter in form of droplets which are usually colored, owing to the presence of carotinoids [89]. In addition, fat cells produce hemoglobin [90] and are supposed to be involved in vitellogenin synthesis [91]. Whether the acidification-induced disturbance in the energy and mass budgets results solely from the increased maintenance costs for ion homeostasis or, additionally, from a reduced assimilation rate (e.g. due suboptimal pH conditions for enzymatic digestion of food in the gut) needs further investigation.

It is important to note that no diapausing eggs occurred in pH 5.5 animals during the six-month experimental period. Obviously, the physiologically demanding condition of pH 5.5 was either not associated with an activation of the stress-signaling cascade responsible for production of male offspring [92,93] or males did not survive until maturity. The pH 6.0 animals, in contrast, were distinguished by the repeated occurrence of parthenogenic eggs with a white cover layer which probably resulted from a fungal infection. Although there are some reports on increased fungal parasitism in daphnids [94,95] and crayfish [82,96] under various stress conditions, it remains to be clarified whether acid stress leads to an increased susceptibility of daphnids to fungal parasites [97].

### The role of acclimation in the tolerance to severe acid stress

**Figure 2:**
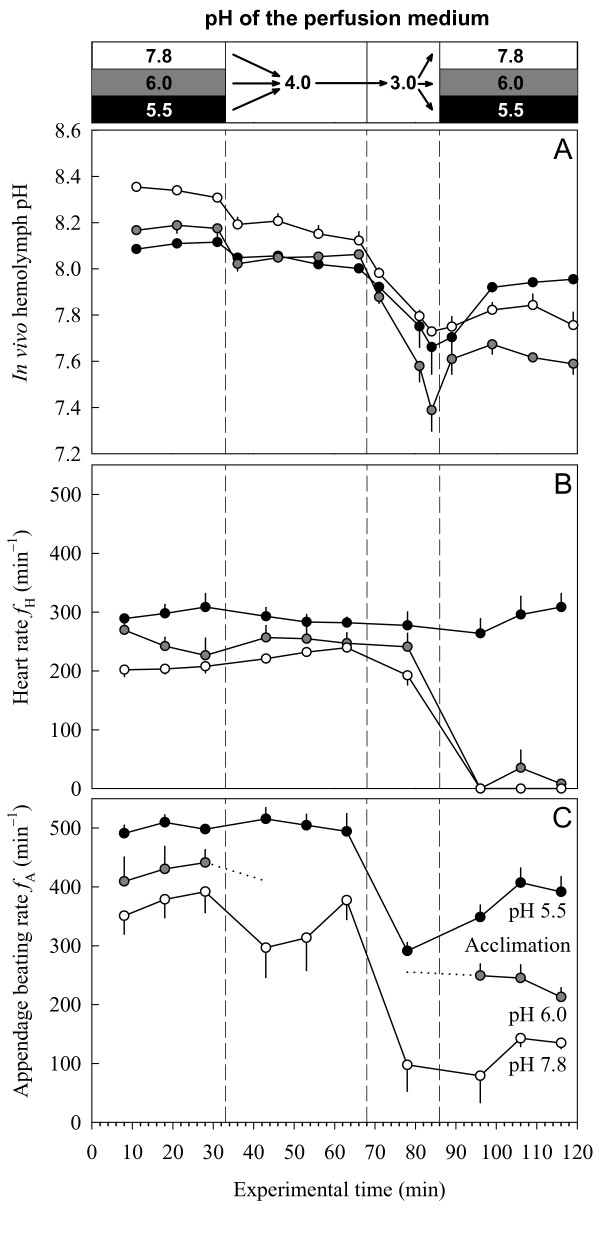
**Tolerance to severe acid stress**. Influence of ambient pH on *in vivo *hemolymph pH (A), heart rate (B) and appendage beating rate (C) of animals acclimated to pH 7.8 (open symbols), pH 6.0 (grey-filled symbols), and pH 5.5 (filled symbols). Data are given as means ± S.E. (*N *= 3–4). Dotted lines indicate a period of irregular limb beating activity. Each acclimation group was exposed to its acclimation pH during the initial and final phases of the experiment.

The tolerance to a short-term exposure to severe acid stress (ambient pH 3–4) was examined in the control and acid-acclimated animals. The animals were initially exposed to their respective acclimation pH before the ambient pH was set to pH 4.0 and then to pH 3.0 (Figure 2). Upon exposure to ambient pH 4.0, all groups experienced an acidosis, but were able to stabilize their extracellular pH at a level 0.1–0.2 pH units below the respective pre-exposure value (Figure 2A). This response was caused by a 'metabolic acid load' of 7.0 meq L^-1 ^(pH 7.8 animals), 4.5 meq L^-1 ^(pH 6.0 animals), and 1.3 meq L^-1 ^(pH 5.5 animals). While the *f*_H _remained unaffected in all groups (Figure 2B), diverging responses were found in *f*_A _(Figure 2C). The *f*_A _response spectrum comprised a transient depression in pH 7.8 animals, an irregular beating behavior in pH 6.0 animals, and a sustained beating activity in pH 5.5 animals.

During the subsequent 18-min exposure to ambient pH 3.0, the pH homeostasis collapsed in all acclimation groups. The extracellular pH showed a progressive decline (Figure 2A), which corresponded to a net flux of acidic equivalents from the ambient medium into the hemolymph of 32 meq L^-1 ^h^-1 ^(pH 7.8 animals), 46 meq L^-1 ^h^-1 ^(pH 6.0 animals), and 16 meq L^-1 ^h^-1 ^(pH 5.5 animals). This massive net influx of acidic equivalents is in line with reports on the breakdown of ion regulation [34,35,98,99]. In *D. magna*, severe acid stress resulted in a 60–70% inhibition of the unidirectional sodium influx and a 130% increase in sodium outflux [34]. Within one hour, these animals lost 30–50% of their body sodium. The whole-body sodium concentration of *D. magna *is 26–41 mmol (kg wet mass)^-1 ^[35,98,100,101], assuming a wet-to-dry mass ratio of 10:1 [102]. This whole-body concentration is consistent with a hemolymph concentration of 65 mM sodium [103], taking into account that the hemolymph comprises 60% of the body volume [102] and that the extracellular fluid contains the main portion of whole-body sodium. The estimated net efflux of sodium (20–30 mmol L^-1 ^h^-1^) from the hemolymph of *D. magna *compares well with the net influx of acidic equivalents into the hemolymph of *D. pulex *(32 meq L^-1 ^h^-1 ^in control animals). This shows that the disturbance in acid-base balance mirrors the disturbance in ion regulation and vice versa.

During the exposure to ambient pH 3.0, the *f*_A _decreased strongly in all groups, whereas *f*_H _remained apparently unaffected (Figure 2B, C). However, the subsequent recovery period revealed a (somewhat delayed) heart arrest and a deformation of heart structure in the pH 7.8 and pH 6.0 animals. Their extracellular pH values during the recovery period remained 0.55 pH units below initial (pre-acid exposure) values. The pH 5.5 animals, in contrast, were able to reduce the difference between the pre- and post-exposure values to 0.16 units. This was the only group which survived the severe-acid test.

Of all acclimation groups, the pH 5.5 animals had the highest tolerance to severe acid stress as indicated by the lowest net influx of acidic equivalents (16 meq L^-1 ^h^-1^). This implies a lower disturbance of extracellular ion regulation in the pH 5.5 animals compared to the other two acclimation groups, which may explain the unique ability to sustain heart-beating activity in the former and heart arrest in the latter. The results of the severe-acid test further suggest that the acclimation to ambient pH 5.5 induced a compensatory increase in active ion transport and/or a reduction in the epithelial permeability for sodium and hydrogen ions. However, the suggested reduction in epithelial ion permeability contrasts with the increased integumental permeability for respiratory gases arising from the impaired carapace formation. It therefore seems that active compensation in ion transport is the more likely defence mechanism.

### Interdependence between acid-base balance and CO_2 _transport

Information on extracellular pH in the heart region and on hemolymph bicarbonate concentration made it possible to determine the local *P*_CO2 _in the pericardial hemolymph. In daphnids, the pericardial space receives hemolymph from the carapace lacuna, which is an important site of gas exchange [36,76], and from the dorsal lacuna, which is fed by the current leaving the intestinal lacuna [36]. In the carapace lacuna, the *P*_CO2 _is low due to the transintegumental diffusion of CO_2 _from the hemolymph into the ambient medium. In the intestinal lacuna (which traverses the body core region), the *P*_CO2 _is high because metabolically produced CO_2 _is released into the hemolymph. The local *P*_CO2 _in the pericardial space therefore assumes an intermediate value that lies between the two *P*_CO2 _extremes. The magnitude of the *P*_CO2 _differences in the circulatory system strongly depends on the presence or absence of a carbonic anhydrase (CA). In the absence of a CA, the interconversion between CO_2 _and  proceeds slowly [104]. For example, a 25 mM bicarbonate solution with a non-bicarbonate buffer value of 2–10 meq L^-1 ^pH^-1 ^needs 3–12 s for a half-change in hydrogen concentration following the abrupt increase in *P*_CO2 _from 5 to 11 kPa [105]. These half-equilibration times apply to 37°C, so even longer would be needed at 20°C. Given a hemolymph circulation time of 21 s in a 2.5 mm *D. magna *at 20°C (cardiac output: 32 nl s^-1 ^[74], hemolymph volume: 680 nl [102], it is clear that hemolymph passage time from the tissues to the respiratory surfaces is too short to bring the uncatalyzed CO_2_+H_2_O↔H^+^+ reaction into *full *equilibrium. Under these circumstances, the main share of metabolically produced CO_2 _would be transported as physically dissolved gas rather than in the chemically combined form as bicarbonate with the consequence of relatively large *P*_CO2 _differences between the loading and unloading sites.

**Figure 3:**
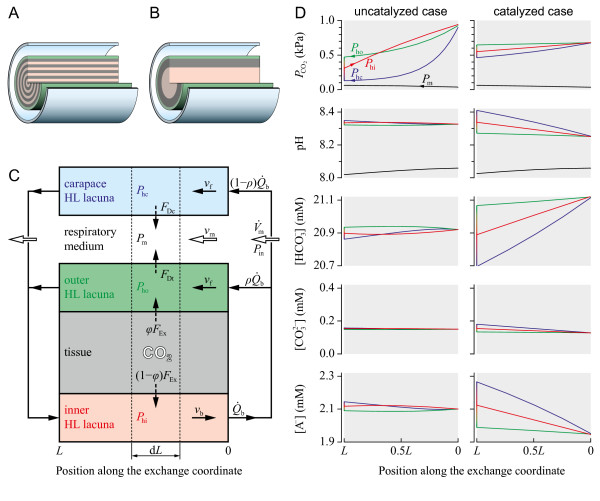
**Modelling and simulation of CO_2 _transport**. (A) Reference topology based on a cylinder-within-tubes arrangement (R. Moenickes, O. Richter and R. Pirow, in preparation). A sector piece was removed to show the alternation of concentric hollow cylinders of tissue (gray) and hemolymph (red, green, blue). (B) Simplified topology with only one tissue layer. This topology is applied in the compartment model. (C) Compartment model of the relevant transport processes. CO_2 _is excreted from the tissue compartment of length d*L *into the inner and outer hemolymph (HL) lacuna at rates of (1-*φ*)*F*_Ex _and *φF*_Ex_. Hemolymph leaving the inner HL lacuna at a volume-flow rate  is distributed between the outer HL lacuna and the carapace HL lacuna. From these compartments CO_2 _diffuses across cuticular barriers into the medium, which flows at a rate of . Indicated are the CO_2 _partial pressures (*P*_hi_, *P*_ho_, *P*_hc_, *P*_m_) and flow velocities (*ν*_b_, *ν*_f_, *ν*_m_) in the hemolymph lacunae and the medium. *P*_in _is the inspiratory *P*_CO2_. (D) Simulation results for the uncatalyzed and catalyzed hydration of CO_2 _for an animal exposed to normal conditions (ambient pH = 8.0, ambient *P*_CO2 _= 0.035 kPa). Acid-base variables are shown for the medium and hemolymph lacunae in relation to the exchange coordinate.

Knowledge about the presence or absence of a CA in the circulatory fluid is therefore fundamental for the understanding of CO_2 _transport and acid-base balance in daphnids. The reported absence of CA activity in the hemolymph of decapod crustaceans [106-109] prompted us to analyse the physiological implications of circulatory CO_2 _transport under uncatalyzed conditions in more detail. Based on established concepts of compartment modelling [110,111] and on own experiences in the simulation of whole-animal oxygen transport in daphnids [71,112], we derived a multi-compartment model of the CO_2 _diffusion-convection-reaction system (Figure 3C) to simulate the transport of CO_2 _from the tissue *via *the hemolymph to the ambient medium. To obtain a pH of 8.334 at the entrance of the inner hemolymph lacuna (Figure 3C), the Krogh constant for the diffusion of CO_2 _in chitin (*K*) was set to 2.10 × 10^-6 ^nmol s^-1 ^mm^-1 ^kPa^-1^. To our knowledge, there are no experimental data in the literature on Krogh's diffusion constant for CO_2 _in chitin. Nevertheless, the chosen *K *value is plausible insofar as it is of the same order of magnitude as Krogh's diffusion constant for O_2 _in chitin (0.95 × 10^-6 ^nmol s^-1 ^mm^-1 ^kPa^-1^) [113]. The similarity in both values seems to contradict the well-known fact that Krogh's diffusion constant for CO_2 _in water and aqueous tissues is 20–25 times higher than that for O_2_, a phenomenon that is explained by the higher capacitance (solubility) coefficient of CO_2 _in *aqueous *media [114]. The cuticle of arthropods, however, is primarily composed of chitin fibers which are embedded in a more or less hydrated protein matrix [115]. Among the different layers (epicuticle, exocuticle, endocuticle), the exocuticle is relatively *dehydrated *[115] and may therefore establish a similar diffusion barrier for O_2 _and CO_2_.

The CO_2 _transport model made it possible to follow the changes in extracellular acid-base variables during a full circulation cycle (Figure 3D, uncatalyzed case). The extracellular *P*_CO2 _in the three hemolymph compartments varies largely between 0.13–0.94 kPa, whereas the extracellular pH remains confined to the narrow range of pH 8.32–8.35. The small changes in bicarbonate concentration (< 0.01 mM) reflect the slow interconversion between CO_2 _and . Compared to bicarbonate, the carbonate and non-bicarbonate buffers show concentration changes in opposite direction as they are involved in the buffering of hydrogen ions arising from the hydration of CO_2 _and subsequent dissociation of carbonic acid.

**Figure 4:**
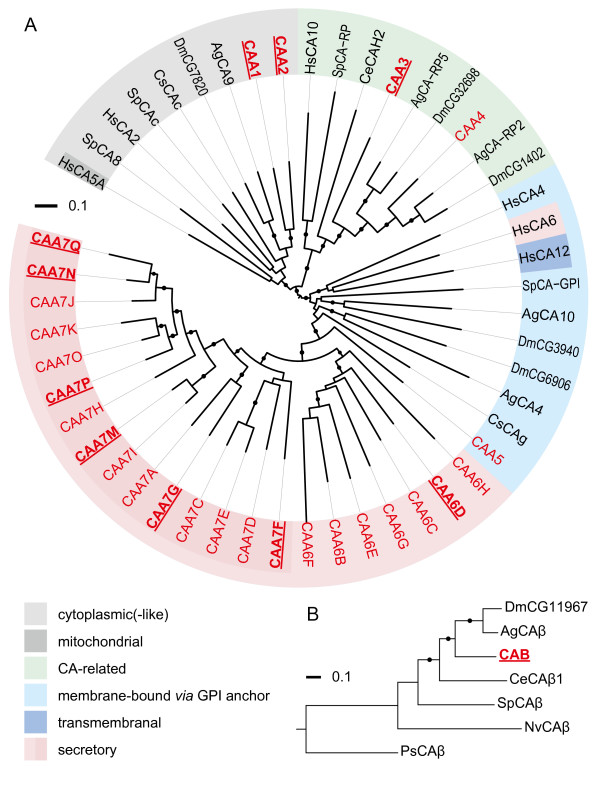
**Classification of CA-like amino acid sequences from *Daphnia pulex***. Phylogenetic trees for selected α-carbonic anhydrases (α-CAs) (A) and β-CAs (B) based on multiple-sequence alignments (Additional files 1 and 2). *D. pulex *sequences are shown in red; underlined labels indicate EST support. Three fragmentary sequences (CAA6A, CAA7B, CAA7L; Table 4) from *D. pulex *were excluded from the alignment. Additionally included were related sequences from the blue crab *Callinectes sapidus *(Cs), *Drosophila melanogaster *(Dm), *Anopheles gambiae *(Ag), *Caenorhabditis elegans *(Ce), sea urchin *Strongylocentrotus purpuratus *(Sp), *Homo sapiens *(Hs), the sea anemone *Nematostella vectensis *(Nv), and *Pisum sativum *(Ps). α-CAs were classified in terms of their putative destination into mitochondrial and cytoplasmic, CA-related, membrane-bound and transmembrane, and secretory proteins. CA-related proteins have lost most of the highly conserved active-site residues. Membrane-associated α-CAs have a C-terminal attachment signal for a glycosylphosphatidylinositol (GPI) anchor which tethers the extracellular protein to the cell membrane [123]. The trees were constructed using the neighbor-joining algorithm. Bootstrap analysis was performed with 1000 replicates (bootstrap values > 800 are indicated by filled circles). For sequence references, see Additional files 1 and 2.

The simulation provides a plausible prediction of the extracellular CO_2 _and pH gradients that would develop in the absence of a hemolymph CA. However, a screening of the *D. pulex *genome database [116,117] unexpectedly revealed 31 genes with CA-like coding sequences (Table 4). These genes belong to two evolutionarily unrelated CA gene families (α-CA and β-CA) [118]. The derived amino-acid sequences were aligned with selected metazoan sequences (Additional files 1 and 2) [119-122] and classified in terms of their putative destination (Figure 4A, B) [123], based on sequence features and the known localization of CAs from crab [124], mosquito [125], and man [118]. The phylogenetic analysis of α-CA sequences showed a distinct separation between mitochondrial and cytoplasmic, CA-related, membrane-bound and transmembrane, and secretory proteins (Figure 4A). Among the 30 α-CAs from *D. pulex *were 25 sequences (CAA6A-H, CAA7A-Q) with an N-terminal signal peptide for secretory export. Seven of these putative extracellular isoforms are currently supported by EST data. So far, nothing is is known about the extracellular target sites. To account for the possibility of CA secretion into the hemolymph, we simulated a second scenario, in which a hemolymph CA accelerates the interconversion between CO_2 _and  by a factor of 10000 [126], which is sufficiently large to establish an equilibrium. Krogh's diffusion constant for CO_2 _in chitin was slightly reduced to 1.30 × 10^-6 ^nmol s^-1 ^mm^-1 ^kPa^-1 ^to obtain a pH of 8.334 at the entrance of the inner hemolymph lacuna (Figure 3C). Compared to the uncatalyzed case, the catalyzed hydration/dehydration of CO_2 _significantly reduced the variations in extracellular *P*_CO2 _to the narrow range of 0.46–0.68 kPa (Figure 3D), indicating that less CO_2 _is transported as physically dissolved gas. Instead, more CO_2 _is transported in the chemically combined form as reflected by the large variation in bicarbonate concentration. Moreover, the extracellular pH varied substantially between the 'prebranchial' value of 8.25 (inner HL lacuna) and the 'postbranchial' value of 8.41 (carapace HL lacuna).

**Table 4:** List of referred carbonic anhydrase-like proteins and gene models from *D. pulex*.

**Symbol**	**Model name**	**Protein ID**	**Reference ID**
CAB	PIR_PASA_GEN_2900003	347880	304414

CAA1	PIR_estExt_fgenesh1_pg.C_80063	442498	222096

CAA2	PIR_estExt_fgenesh1_pg.C_80158	442497	222141

CAA3	PIR_e_gw1.74.6.1	442499	58540

CAA4	PIR_e_gw1.4.553.1	442496	42376

CAA5	PIR_NCBI_GNO_2000180	442477	317362

CAA6A	PIR_1_NCBI_GNO_0400291	442779	311517

CAA6B	PIR_SNAP_00002730	442471	41941

CAA6C	PIR_e_gw1.4.143.1	442472	41941

CAA6D	PIR_PASA_GEN_0400293	442467	305654

CAA6E	PIR_NCBI_GNO_0400294	442475	311520

CAA6F	PIR_e_gw1.4.154.1	442468	42212

CAA6G	PIR_e_gw1.4.906.1	442476	42004

CAA6H	PIR_e_gw1.4.98.1	442478	42484

CAA7A	PIR_PASA_GEN_0400138	442480	305530

CAA7B	PIR_NCBI_GNO_0400455	442481	42005

CAA7C	PIR_SNAP_00002914	442482	none

CAA7D	PIR_SNAP_00002915	442483	42005

CAA7E	PIR_NCBI_GNO_0400456	442484	42005

CAA7F	PIR_PASA_GEN_0400354	442479	305707

CAA7G	PIR_PASA_GEN_3600071	442494	305268

CAA7H	PIR_SNAP_00002923	442485	234865

CAA7I	PIR_NCBI_GNO_0400466	442486	42371

CAA7J	PIR_e_gw1.4.668.1	442487	42371

CAA7K	PIR_SNAP_00002925	442488	234867

CAA7L	PIR_SNAP_00002926	442489	234868

CAA7M	PIR_NCBI_GNO_0400472	442491	221343

CAA7N	PIR_estExt_fgenesh1_pg.C_40469	442490	221343

CAA7O	PIR_NCBI_GNO_0400474	442492	311700

CAA7P	PIR_estExt_Genewise1.C_40740	442493	207081

CAA7Q	PIR_estExt_fgenesh1_pg.C_400146	442495	225703

Given are the protein names, gene model names and protein identification numbers of those loci which were annotated in the present study. Protein IDs indicate manually curated gene models that may differ from those contained in the 'Filtered Models v1.1' set (released by JGI in July 2007) [117]. The Reference ID can be used to retrieve the corresponding models from the Filtered Models set v1.1.

The two simulated scenarios represent a coherent description of the physiological implications arising from the presence or absence of a CA in the hemolymph of *D. pulex*. The selected values for the global adjustment parameter *K *remain within reasonable bounds that made it impossible to put more weight to one of the two models. Nevertheless, the predicted extracellular *P*_CO2 _and pH gradients represent a working hypothesis that will be tested in subsequent experiments. pH imaging techniques, for example, should have the resolution power to detect a spatial *in vivo *gradient as large as 0.1–0.2 pH units to verify or falsify the assumption of CA activity in the circulatory system of *D. pulex*. Further *in vivo *experiments may include the application of a strong diffusible CA inhibitor or the microinjection of an exogenous CA [127].

## Conclusion

Chronic acid exposure induced pronounced effects in extracellular pH, bicarbonate concentration and CO_2 _partial pressure, as well as in circulation, ventilation and energy metabolism. Compensatory changes in extracellular non-bicarbonate buffering capacity and the improved tolerance to severe acid stress indicated the activation of defense mechanisms. The physiological changes were associated with an impairment of carapace formation and with reductions in reserve materials and reproduction. Mechanistic analyses of the interdependence between extracellular acid-base balance and CO_2 _transport led to the identification and classification of 31 carbonic anhydrase isoforms which are encoded in the genome of *D. pulex*. The multitude of physiological information that can be acquired from these transparent crustaceans *via *optical techniques underlines the great advantage of *Daphnia pulex *as a model system for environmental studies. Proteomic analyses are underway to identify the molecular mechanisms and target genes involved in *Daphnia's *responses to a variety of environmental stresses including freshwater acidification.

## Methods

### Acclimation conditions

Animals were raised at 20°C in aerated M4 medium [128] under three different pH conditions at a 16 h:8 h L:D photoperiod. The control condition (7.8 ± 0.2, mean ± variation range) was manually adjusted twice a week using 0.005 M H_2_SO_4 _and 0.01 M NaOH. The pH 6.0 ± 0.1 condition was established by adding 5 mM MES buffer (2-morpholinoethansulfonic acid) to the medium. pH 5.5 ± 0.05 was maintained by a pH-Stat, which was equipped with a pH electrode (N 6000; Schott-Geräte GmbH, Mainz, Germany) and which controlled the addition of 0.05 M H_2_SO_4 _delivered by a peristaltic pump (Gilson ABIMED, Villiers, France).

The pH 7.8 and pH 6.0 animals were cultured in 2 L glass beakers (containing 1.8 L medium) at a density of 25–50 individuals (juveniles plus adults) per vessel. The pH 5.5 animals were kept in a 20 L glass aquarium (containing 8 L medium) at a density of 100–200 individuals. Surplus offspring were sorted out twice a week. Given the case of appearance, females with ephippia and males were sorted out, so that parthenogenesis and clonal reproduction was maintained. Animals were fed *ad libitum *with *Desmodesmus subspicatus *(final concentration: 15.5 × 10^4 ^cells per ml culturing medium) six times a week. To minimize the influence of algae on medium pH [129], sedimented food material was removed once (glass aquarium) or twice (glass beaker) a week. During this procedure, any algal surface buildup was removed by scrubbing the enclosures, and at least half of the medium was exchanged by fresh medium.

### Analysis of hemolymph buffer curves

Hemolymph samples (0.2–1 μl per animal) were drawn as described elsewhere [73] and collected in ice-cooled 500 μl reaction vials. The pooled hemolymph (30–100 μl) was filtered (cellulose acetate syringe filters, 0.45 μm pore size; Nalgene, Rochester, NY), shortly centrifuged to remove any air bubbles, and finally kept on ice. Hemolymph buffer curves were measured with a micro-pH-electrode (MI-4152; Microelectrodes Inc., Bedford, U.S.A.) in a gas diffusion chamber [130] at 20°C. The pH electrode was linked to a pH-meter (MP 230, Mettler Toledo, Swiss) which transferred the data to a computer. Traceable NIST standard reference buffers (pH 6.88 and pH 9.23 at 20°C, type number: L 4798; Schott-Geräte GmbH) were used for calibration. Hemolymph samples of 5–10 μl were equilibrated with humidified gas mixtures of different CO_2 _partial pressure (*P*_CO2 _= 0.135–5.50 kPa). The gas mixtures were prepared from highly pure nitrogen (> 99.996%) and carbon dioxide (99.995%; Air Liquide, Düsseldorf, Germany) using a gas mixing pump (2 M 303/a-F Wösthoff oHG Bochum, Germany).

For analysis, the hemolymph of *Daphnia *was considered as a binary buffer system consisting of the carbonate system and a monoprotic non-bicarbonate buffer (HA ↔ H^+ ^+ A^-^). The dependence of pH on *P*_CO2 _for such a system is described by the following balance equation [131]

(1)

where {H^+^} is 10^-pH^, *K'*_w _(= 10^-14 ^M) is the dissociation equilibrium constant of water, and SID represents the strong ion difference [132]. *C*_A _and *K'*_A _are the concentration and dissociation equilibrium constants of the non-bicarbonate buffer, whereas *K'*_1 _and *K'*_2 _represent the first and second dissociation equilibrium constants of the carbonate system. The physical solubility of CO_2 _in hemolymph (α_CO2 _= 0.3682 mmol l^-1 ^kPa^-1^) was calculated according to a thermodynamic model [133,134], assuming a sodium concentration of 58 mM and a solution density of 1 g l^-1^. Operational p*K'*-values of the carbonate system (p*K'*_1 _= 6.325 ± 0.002, p*K'*_2 _= 10.47 ± 0.09; means ± S.E.) were determined from standard bicarbonate solutions (4, 8, 16 mM NaHCO_3 _plus 50 mM NaCl) using three equilibration steps (*P*_CO2 _= 0.13, 0.50, 2.0 kPa) (Additional file 3). These standard bicarbonate solutions were a valid representation of *Daphnia *hemolymph in terms of ionic strength which, besides protein concentration, affects the α_CO2 _and the p*K'*-values [131]. The influence of protein concentration on α_CO2 _was negligible because the proteins in *Daphnia *hemolymph occupy less than 1% of hemolymph volume.

Given the *P*_CO2_-pH data, parameter values for SID, *C*_A _and *K'*_A _were obtained by nonlinear least-squares data fitting. The analytical procedure additionally contained a correction for incomplete hemolymph equilibration at the lowest *P*_CO2 _step (Additional file 3). The concentrations of bicarbonate and carbonate are given by

(2)

and

(3)

The appropriateness of the operational parameter values for the calculation of bicarbonate was validated by the direct measurement of total CO_2 _in *Triops cancriformis *hemolymph (R. Pirow, unpublished data), whose ionic strength is comparable to that of *Daphnia *hemolymph. The non-bicarbonate buffer value (*β*_A_) was obtained from [131,135]

(4)

The buffer values of bicarbonate (*β*_B_) and carbonate (*β*_C_) were determined for the open-system condition [135,136], under which the hemolymph *P*_CO2 _is assumed to be held constant *in vivo *(as by the control of ventilation) [136]:

(5)

and

(6)

Finally, the concentration of acidic equivalents added to the hemolymph (ΔH^+^, 'metabolic acid load') [78] during acute exposure to severe acid stress was obtained from

(7)

where the subindices 1 and 2 refer to the concentrations before and during the exposure.

### Microfluorometric set-up

Fluorescence measurements were performed with an inverted microscope (Axiovert 10, Carl Zeiss, Oberkochen, Germany) equipped with a monochromatic illumination system (T.I.L.L. Photonics, Planegg, Germany) and an imaging spectrograph (SpectraPro-275I, Acton Research Corporation, Acton, MA, USA). A 10 × objective (Plan Neofluar, Zeiss) was used for all experiments. The fluorescence light was transmitted by a quartz fibre-optic light guide to the imaging spectrograph, which was equipped with a thermoelectrically-cooled (-10°C) CCD camera (HLS 1024/64bi; Proscan elektronische Systeme GmbH, Lagerfeld, Germany) containing a highly-sensitive (back-thinned), 16-bit CCD chip (1024 × 58 pixels; S7031-1006, Hamamatsu Photonics, Herrsching am Ammersee, Germany). A CCD exposure time of 2 min was used for spectrum acquisition. Fluorescence emission spectra were smoothed with a span of 30 nm.

### Calibration of cSNARF-1

A stock solution of 70-kDa dextran-coupled cSNARF-1 (D-3304, Molecular Probes, Inc., Oregon, USA) was prepared by dissolving 5 mg lyophilized dye in 250 μl sterile-filtered Milli-Q water (Millipore, Schwalbach, Germany). The stock solution was 1:20 diluted with a 10 mM NaHCO_3 _solution containing 50 mM NaCl. Given a conjugation ratio of 3–8 chromophore groups per dextran particle (Molecular Probes product information, 2003), the average concentration of cSNARF-1 in the calibration solution was 80 μM. Using the diffusion chamber and the microfluorometric set-up described above, a 10 μl sample was equilibrated with gas mixtures of different *P*_CO2 _(0.135–5.50 kPa). At the end of each equilibration step, a fluorescence spectrum and the sample pH were measured.

**Figure 5:**
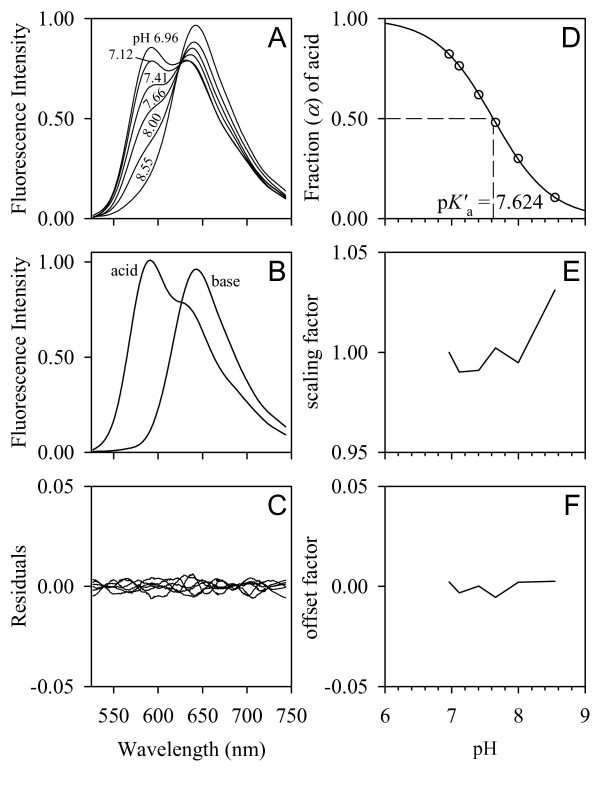
**Calibration of cSNARF-1**. Fluorescence emission spectra of a bicarbonate-buffered cSNARF-1 solution were acquired at various pH values (A). Fluorescence excitation was at 475 nm. Reiterative least-squares spectral resolution [137] (Additional file 4) was applied to the data to extract the spectra of the acid/base forms (B) and the p*K'*_a _value of cSNARF-1. The underlying model that relates the fraction of acid *α *to pH (D) is given by *α *= {H^+^}/(*K'*_a _+ {H^+^}). The optimum p*K'*_A _value of 7.624 was reiteratively obtained by using the Nelder-Mead simplex algorithm [144]. The inclusion of an additive offset factor (E) and multiplicative scaling factor (F) into the optimization corrected for variations in CCD dark current, excitation light intensity, sample shape, and fluorophore concentration. The residuals (C) represent 'unexplained' spectral information (noise).

The CO_2 _titration of a bicarbonate-buffered cSNARF-1 solution containing additionally 50 mM NaCl had the advantage of calibrating the pH-sensitive dye in a chemical environment whose ionic composition is similar to that of *Daphnia *hemolymph (see Discussion). However, the chosen CO_2 _partial pressures were not sufficient to achieve pH extremes which could shift the dye into the fully protonated (acid) and deprotonated (base) forms (Figure 5A). Reiterative least-squares spectral resolution (Additional file 4) [137] was therefore employed to recover the spectra of the acid/base forms (Figure 5B) and the p*K'*_a _value of cSNARF-1. The calibration yielded a p*K'*_a _of 7.624 (Figure 5D).

### *In vivo *measurements

Adult females with a carapace length of 1.9–2.6 mm and parthenogenetic embryos of developmental stage 2 [138] were used. Animals were immobilized as described [73]. For cSNARF-1 microinjection, small glass capillaries (GB 120 F10, Science Products GmbH, Hofheim, Germany) were thinly drawn out with a micropipette puller (model 77; Sutter Instruments, Novato, CA, USA). 2 μl cSNARF-1 stock solution (1.57 mM) were loaded into the micropipette. The solution was microinjected (Transjector 5246; Eppendorf, Hamburg, Germany) through the basal joint membrane of one of the large antennae into the hemolymph space. The injection was followed using a stereomicroscope (SZH-ILLK; Olympus GmbH, Hamburg, Germany). After 2–6 hours of recovery in nutrient-free medium, the animal was transferred into a perfusion chamber as described elsewhere [73]. The flow rate of the perfusion medium was maintained at 5.5 ml min^-1 ^using a peristaltic pump (MCP Standard ISM 404; Ismatec SA, Glattbrugg, Swiss). The initial pH of the perfusion medium corresponded to the acclimation pH of the animals. During the experiment, the animal was exposed to a stepwise variation in ambient pH using the following sequence: initial (acclimation) pH (33 min), pH 4.0 (35 min), pH 3.0 (18 min), and acclimation pH (34 min). All perfusion media were buffered using 5 mM HEPES (pH 7.8), 5 mM MES (pH 6.0), or 5 mM citrate (pH 5.5, 4.0, 3.0). The medium pH was continuously controlled using a pH electrode (N 6000). During the experiment, the fluorescence-spectrum acquisition alternated with the acquisition of video images of the animal under infrared transillumination. From these video sequences, the heart rate and appendage beating rate was determined by digital motion analysis as described elsewhere [73].

### Analysis of *in vivo *cSNARF-1 spectra

**Figure 6:**
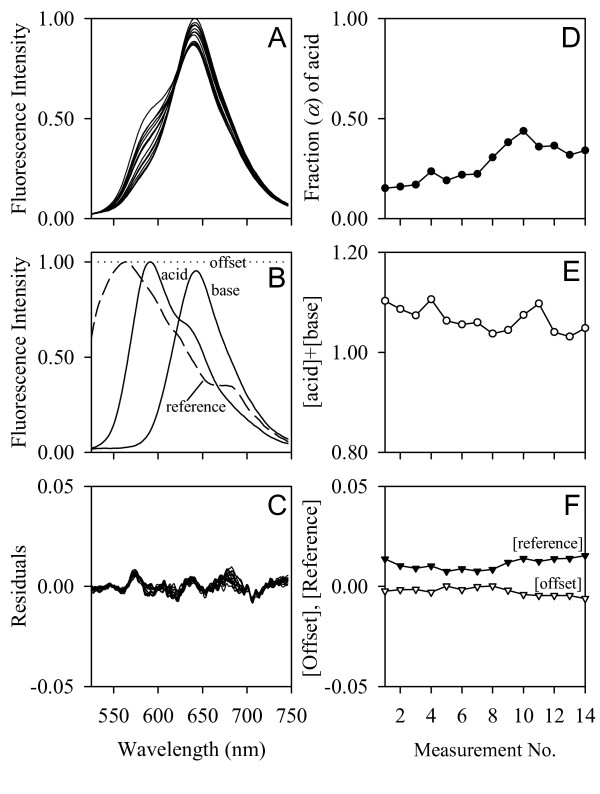
**Analysis of *in vivo *cSNARF-1 spectra**. Example *in vivo *spectra (A) from a pH 7.8 acclimated *Daphnia pulex *exposed to ambient pH 7.8-3.0. The corresponding pH values were retrieved by a multicomponent analysis [139] (Additional file 4), which determines the composition of a mixture of components, given that the spectrum of each component is known. The component spectra (B) comprised the *in vitro *spectra of the acid/base forms of cSNARF-1 (measured in *Daphnia *hemolymph), a reference (autofluorescence) spectrum from non-injected animals, and an offset (background) spectrum. The multicomponent analysis yielded the fraction of the acid form (D), the relative chromophore (acid plus base) concentration (E), as well as the contributions of the reference and offset signals (F). The residuals (C) represent spectral information that could not be explained by the component signals.

cSNARF-1 fluorescence spectra were obtained from the hemolymph space around the heart region. Since all tested animals were in a fasting state, the *in vivo *spectra did not contain any noticeable contributions from ingested autofluorescing algae which, if present, would have seriously affected the pH determination. The excellent quality of the *in vivo *cSNARF-1 spectra (Figure 6A) made it possible to determine the *in vivo *pH with high precision using multicomponent analysis (Additional file 4) [139]. Since the *in vivo *spectra could not be fitted by the calibration spectra (Figure 5B), probably due to a calibration-inherent distortion of the acid spectrum around 600–700 nm, new acid/base spectra of cSNARF-1 were measured in *Daphnia magna *hemolymph. The hemolymph samples were acidified by equilibration with 100% CO_2 _gas and basified by the addition of NaOH under CO_2_-free gas conditions (100% N_2_). As a modification to the calibration experiment, the micro-pH-electrode was not inserted into the hemolymph samples to avoid any optical interferences. The obtained acid/base spectra (Figure 6B) were finally scaled to the peak-to-peak ratio of the calibration spectra (Figure 5B). The multicomponent analysis determined the fractional contribution (*α*) of the acid form of cSNARF-1 to the *in vivo *spectra. The pH was finally calculated from *α *and the p*K'*_a _value of cSNARF-1 (Figure 6D) according to

(8)

### Respiration measurements

The oxygen consumption rate () of a group of 3–4 animals (2.0–3.3 mm body length) carrying parthenogenetic embryos of developmental stage 1–2 [138] was measured at 20°C as described elsewhere [74]. The respiratory medium consisted of M4 medium containing 10 mM buffer (HEPES: pH 7.8, MES: pH 6.0, citrate: pH 5.5). Tetrazyclin and Streptomycin (12.5 mg l^-1 ^each) was added to reduce bacterial respiration. The specific oxygen consumption rate was obtained by dividing the whole-animal oxygen consumption rate by the cubic body length.

### Modelling of whole-animal CO_2 _transport

A topological model was derived from a geometric concept on convective-diffusive oxygen transport in daphnids [71,112]. In this concept, the animal's complex body is reduced to a cylindrical trunk which is wrapped by a hollow cylinder representing the carapace (Figure 3A). The carapace is a double-walled, hollow structure that is perfused with hemolymph. The hollow-cylindric space between the carapace and the trunk is occupied by the respiratory medium. As a simplification of the reference model (Figure 3A), the present model is composed of only five subdomains (Figure 3B). These are the inner hemolymph lacuna, a single tissue layer, the outer hemolymph lacuna, the respiratory medium, and the carapace hemolymph lacuna, as outlined in the conceptual overview of the compartment model (Figure 3C). Each subdomain has a total length *L *and is divided into *N *compartments of length d*L*.

The processes operating within each compartment and in between adjacent/connected compartments include (i) the excretion of CO_2 _from tissue into hemolymph, (ii) the CO_2 _hydration and acid-base reactions in hemolymph and medium, (iii) the convective transport of reaction species, and (iv) the diffusive transport of CO_2 _across cuticular barriers. A mathematical formulation of the physico-chemical processes is given for a single compartment of the outer hemolymph lacuna. For compartments of other subdomains, equations can be derived in an analogous manner.

(i) The rate (nmol s^-1^) at which CO_2 _is excreted from a tissue compartment of thickness d*L *into the outer hemolymph lacuna is *φF*_ex _with

(9)

where  is the whole-animal CO_2 _production rate. The factor *φ *is the fraction of excreted CO_2 _that is released into the outer hemolymph lacuna. The remaining fraction (1-*φ*) is received by the inner hemolymph lacuna.

(ii) The hydration and subsequent dissociation of CO_2_, its combination with OH^-^, and the dissociation of bicarbonate and the non-bicarbonate buffer HA are given by



The lower and upper-case *k*s represent kinetic and thermodynamic constants (Table 5), whereas μ is the factor by which the uncatalyzed interconversion between CO_2 _and  is accelerated in the presence of a carbonic anhydrase. The turnover rates (mol L^-1 ^s^-1^) of the forward and backward reactions are defined as

**Table 5:** Parameter values of the CO_2 _transport model.

**Symbol**	**Value**	**Unit**	**Description**
*L*	2.38	mm	Length of exchange coordinate

*A*_ca_	7.57	mm^2^	Exchange surface area of the inner carapace cuticle

*A*_tr_	5.34	mm^2^	Exchange surface area of the trunk cuticle

Δ*x*_ca_	0.001	mm	Thickness of the inner carapace cuticle

Δ*x*_tr_	0.002	mm	Thickness of the trunk cuticle

	0.022	mm^3 ^s^-1^	Perfusion rate

	0.7	mm^3 ^s^-1^	Medium flow rate

*ν*_b_	0.168	mm s^-1^	Hemolymph flow velocity, backward direction

*ν*_f_	0.149	mm s^-1^	Hemolymph flow velocity, forward direction

*ν*_m_	1.8	mm s^-1^	Medium flow velocity

	0.0071	nmol s^-1^	Whole-animal CO_2 _production rate

	0.3682	nmol mm^-3 ^kPa^-1^	Physical solubility of CO_2 _in medium and hemolymph

*K*	2.10 × 10^-6^	nmol s^-1 ^mm^-1 ^kPa^-1^	Krogh's diffusion constant for CO_2 _in chitin

*K'*_1_	10^-6.325^	M	Dissociation equilibrium constant of CO_2_

*K'*_2_	10^-10.47^	M	Dissociation equilibrium constant of

*K'*_A_	10^-8.18^	M	Dissociation equilibrium constant of the NB buffer

*K'*_w_	10^-14^	M	Dissociation equilibrium constant of water

*k*_1_	0.022	s^-1^	Rate constant for CO_2 _hydration

*k*_2_	10^10^	M^-1 ^s^-1^	Rate constant for the protonation of

*k*_3_	5500	M^-1 ^s^-1^	Rate constant for the reaction of CO_2 _with OH^-^

*k*_-3_	1.1 × 10^-4^	s^-1^	Rate constant for: → CO_2 _+ OH^-^

*k*_4_	10^10^	M^-1 ^s^-1^	Rate constant for the protonation of the NB buffer

μ	1		Acceleration factor for CO_2_/ interconversion

ρ	0.5		Fraction of entering the outer HL lacuna

φ	0.2		Fraction of CO_2 _excreted into the outer HL lacuna

*γ*_H_	0.797		Hydrogen activity coefficient

*C*_A_	3.6	nmol mm^-3^	Concentration of the NB buffer in the hemolymph

*P*_in_	0.035	kPa	Inspiratory CO_2 _partial pressure

pH_in_	8.0		pH of the inspired medium

[]_in_	0.6	nmol mm^-3^	Bicarbonate concentration of the inspired medium

These parameter values were used to generate the profiles in acid-base variables shown in Figure 3D. The values for *K *and μ refer to the uncatalyzed case in the absence of a carbonic anhydrase in the hemolymph. The catalyzed case was derived from this parameter setting by two adjustments (μ = 10000, *K *= 1.30 × 10^-6 ^nmol s^-1 ^mm^-1 ^kPa^-1^). NB = non-bicarbonate.

(10)

(11)

(12)

(13)

(14)

(15)

(16)

(17)

The hydrogen activity, {H^+^}, was calculated from hydrogen concentration as {H^+^} = *γ*_H _[H^+^]. The H^+ ^activity coefficient (*γ*_H _= 0.797) was determined for an ionic strength of 0.06 at 20°C using the Güntelberg approximation [140].

(iii) The net convective mass flow (nmol s^-1^) of each reaction species (X = H^+^, CO_2_, , , HA, A^-^) from the upstream compartment into the compartment in consideration is

(18)

where [X] and [X]_upstream _represent the species concentrations in the compartment in focus and in the upstream compartment. The factor *ρ *is the fraction of total hemolymph flow () that is fed into the outer hemolymph lacuna.

(iv) The rate (nmol s^-1^) of transcuticular CO_2 _diffusion, which depends on the difference in CO_2 _partial pressure between the outer HL lacuna (*P*_ho_) and the medium (*P*_m_), is defined as

(19)

*K *is Krogh's diffusion coefficient, whereas Δ*x*_tr _and *A*_tr_d*L*/*L *represents the thickness and surface area of the cuticular barrier at the hemolymph/medium interface.

The temporal changes in the concentration of all reaction partners for the specified compartment of volume *V *(= *ρ*d*L*/ν_f_) are expressed as

(20)

(21)

(22)

(23)

(24)

(25)

Parameter values (Table 5) related to geometry, convection and respiration were obtained from a reference model (R. Moenickes, O. Richter and R. Pirow, in preparation). All perfusion-related parameter values were set to 50% of the reference values to take the low heart rates of animals from the present study into account. The rate constants for the reaction of CO_2 _with H_2_O and OH^- ^at 20°C were obtained from [141]. The acceleration factor (μ) was set to 10000 [126], which is sufficiently large to attain an equilibrium in the CO_2_+H_2_O↔H^+^+ reaction. The protonation rate constant for the carbonate and the non-bicarbonate buffer was assumed to be of the magnitude of 10^10 ^M^-1 ^s^-1 ^[111]. The dissociation equilibrium constants of all reaction species as well as the physical solubility of CO_2 _were taken from the present study. An operational value for Krogh's diffusion constant (*K*) for CO_2 _in chitin was chosen as such that the pH at the entrance of the inner hemolymph lacuna (Figure 3C) assumed a value of pH 8.334 under steady-state conditions. The cuticular barrier was assumed to be impermeable for all reaction species except CO_2_, and the medium compartment lacked a non-bicarbonate buffer. The initial conditions for the hemolymph were pH 8.334 and 0.556 kPa *P*_CO2_. The initial conditions of the medium compartment were set to the properties of the inspired medium (pH 8.06 and 0.035 kPa *P*_CO2_). A number of *N *= 50 compartments was chosen per subdomain. Starting with the initial conditions, the model status was allowed to evolve until quasi steady-state conditions (relative concentration changes < 10^-6^) were reached.

### Statistics and Numerics

If not stated otherwise, data are expressed as means ± standard error, with *N *indicating the number of independent measurements. Differences in a physiological variable among the acclimation groups were checked using a one-way analysis on variance (ANOVA) or the Kruskal-Wallis test, depending on whether the data passed the normality test and the equal variance test. Statistical differences were considered as significant at *P *< 0.05. Multiple pairwise comparisons against the control (pH 7.8) group were performed using the Holm-Sidak test or Dunn's method, using an experimentwise significance level of 0.05. All statistical analyses were performed using SigmaStat (version 3.1; SPSS Inc.).

Numerical problems were solved in Matlab 7.0 (MathWorks, Inc.). The 'lsqnonlin' function (optimization toolbox) was used to fit the model in equation 1 to the *P*_CO2_-pH data. The uncertainty in the calculation of *P*_CO2_, given the pH and the calibration buffer curve, was determined by a nonlinear algorithm [142,143]. The 'rlowess' function (curve-fitting toolbox) was applied for the smoothing of spectra. In-built functions for matrix operations (including that for the calculation of the Moore-Penrose pseudoinverse) were used to implement the reiterative least-squares spectral resolution [137] and the multicomponent analysis [139] (Additional file 4), whereas the 'fminsearch' function (optimization toolbox) provided the Nelder-Mead simplex algorithm [144]. The nonlinear system of ordinary differential equations (ODEs) was numerically solved using the 'ode15s' solver for stiff problems.

### Annotations, sequence alignments and phylogenetic analysis

The *D. pulex *genome database was screened for carbonic anhydrase-like sequences by a keyword search in the automatically-created annotations and by a 'blastp alignment search' of the Dappu v1.1 gene builds (July, 2007) [117]. All gene models containing carbonic anhydrase-like sequences were manually curated and annotated (Table 4). The derived amino-acid sequences were classified using the conserved domain database (CDD) and search engine v2.13 [145,146]. Homolog sequences from other organisms were retrieved using the blastp algorithm [147]. All sequences were checked for the presence of N-terminal signal peptides using the SignalP V3.0 server [148-150]. Potential GPI-anchor sites were identified by GPI-SOM [151,152], the big-PI Predictor [153,154] and FragAnchor [155,156]. Multiple-sequence alignments were performed using the T-Coffee algorithm [157-159] and displayed with ESPript [160,161]. Phylogenetic trees were constructed using the neighbor-joining algorithm [162] and a bootstrap analysis with 1000 replicates. Trees were visualized using iTOL [163,164].

## Abbreviations

*A*_ca_: exchange surface area of the inner carapace cuticle; *A*_tr_: exchange surface area of the trunk cuticle; *C*_A_: concentration of the non-bicarbonate buffer; *f*_A_: appendage beating rate; *f*_H_: heart rate; *F*_Dc_: rate of CO_2 _diffusion across the inner carapace cuticle; *F*_Dt_: rate of CO_2 _diffusion across the trunk cuticle; *F*_Ex_: CO_2 _excretion rate; *F*_X_: net convective mass flow (X = H^+^, CO_2_, , , HA, A^-^); *K*: Krogh's diffusion constant for CO_2 _in chitin; *K'*_1_: first dissociation equilibrium constant of the carbonate system; *K'*_2_: second dissociation equilibrium constants of the carbonate system; *K'*_A_: dissociation equilibrium constant of the non-bicarbonate buffer; *K'*_a_: dissociation equilibrium constant of cSNARF-1; *K'*_w_: dissociation equilibrium constant of water; *k*_1_, rate constant for CO_2 _hydration; *k*_2_: rate constant for the protonation of ; *k*_3_, rate constant for the reaction of CO_2 _with OH^-^; *k*_-3_: rate constant for the dissociation of  into CO_2 _and OH^-^; *k*_4_: rate constant for the protonation of the non-bicarbonate buffer; *L*: length of the exchange coordinate; d*L*: compartment thickness; : whole-animal CO_2 _production rate; : volume-specific O_2 _consumption rate; *P*_CO2_: CO_2 _partial pressure; *P*_in_: inspiratory CO_2 _partial pressure; *P*_hi_: CO_2 _partial pressure in the inner hemolymph lacuna; *P*_ho_: CO_2 _partial pressure in the outer hemolymph lacuna; *P*_m_: CO_2 _partial pressure in the medium; pH_in_: pH values of the inspired medium; : perfusion rate; *R*_Y_: turnover rates (Y = 1, -1, 2, -2, 3, -3, 4, -4); SID: strong ion difference; *V*: compartment volume; : medium flow rate; *ν*_b_: hemolymph flow velocity in backward direction; *ν*_f_: hemolymph flow velocity in forward direction; *ν*_m_: medium flow velocity; *α*: fraction of acid; *α*_CO2_: physical solubility of CO_2 _in water and hemolymph; *β*_A_: non-bicarbonate buffer value; *β*_B_: bicarbonate buffer value; *β*_C_: carbonate buffer value; *β*_T_: total buffer value; ΔH^+^: metabolic acid load; Δ*x*_ca_: thickness of the inner carapace cuticle; Δ*x*_tr_: thickness of the trunk cuticle; *γ*_H_: H^+ ^activity coefficient; ρ: fraction of total hemolymph flow entering the outer hemolymph lacuna; φ: fraction of CO_2 _excreted into the outer hemolymph lacuna; μ: acceleration factor for the interconversion between CO_2 _and .

## Authors' contributions

AKW and RP conceived the study, carried out the methodical developments, and wrote the manuscript. AKW carried out the experiments. RP implemented the numerical tools, developed and implemented the CO_2 _transport model, and annotated the carbonic anhydrase genes. Both authors read and approved the final manuscript.

## Supplementary Material

Additional file 1 **Multiple sequence alignment of α-carbonic anhydrases**. The α-CA sequences are divided into four groups according to similarity. Residues strictly conserved have a red background, residues well conserved within a group according to a Risler matrix [122] are indicated by red letters. Residues conserved between groups are boxed. Secondary structure elements of three human α-CAs are shown in blue on the top: helices with squiggles, beta strands with arrows, alpha and beta turns with TTT and TT letters. The numbering refers to HsCA2. Amino acid residues involved in zinc-binding and in the hydrogen-bonding network are indicated by red triangles. Yellow and orange backgrounds indicate mitochondrial targeting peptide or predicted signal peptides for secretory export. Pink and green backgrounds signify a transmembrane domain or potential glycosylphosphatidylinositol (GPI) anchor sites. *Daphnia pulex *sequences are indicated by red labels. Additionally included were related sequences from the blue crab *Callinectes sapidus *(Cs), *Drosophila melanogaster *(Dm), *Anopheles gambiae *(Ag), *Caenorhabditis elegans *(Ce), the sea urchin *Strongylocentrotus purpuratus *(Sp), and *Homo sapiens *(Hs). Sequences were aligned using the T-Coffee algorithm [158] and displayed with ESPript [120,161]. Sequence references, protein data bank (PDB) codes and NCBI accession numbers: *Callinectes *[124], *Drosophila *[119], *Anopheles *[125], HsCA2 (1CA2), HsCA4 (1ZNC), HsCA5A (NP_001730), HsCA6 (P23280), HsCA10 (AAH29865), HsCA12 (1JCZ), CeCAH2 (Q18932), SpCA8 (XP_795365), SpCAc (XP_782997), SpCA-RP (XP_784796), SpCA-GPI (XP_796525). Click here for file

Additional file 2 **Multiple sequence alignment of β-carbonic anhydrases**. Numbering and the secondary structure elements on the top refer to the β-CA from *Pisum sativum *(PsCAb) [121]. The other sequences are from *Daphnia pulex *(CAB), *Drosophila melanogaster *(DmCG11967), *Anopheles gambiae *(AgCAb), *Caenorhabditis elegans *(CeCAb1), sea urchin *Strongylocentrotus purpuratus *(SpCAb), and the sea anemone *Nematostella vectensis *(NvCAb). A column is framed in blue if more than 70% of its residues are similar according to physico-chemical properties. Similar residues are indicated by red letters; strictly conserved residues have a red background. Secondary structure elements are presented as follows: helices with squiggles, beta strands with arrows, alpha and beta turns with TTT and TT letters. Amino acid residues involved in zinc and substrate binding are indicated by red and blue triangles. Sequences were aligned using the T-Coffee algorithm [158] and displayed with ESPript [120,161]. Protein data bank (PDB) code and NCBI accession numbers: PsCAb (2EKJ), DmCG11967 (NP_649849), AgCAb (XP_563117), CeCAb1 (NP_741809), SpCAb (XP_786120), NvCAb (XP_001632619). Click here for file

Additional file 3 **Determination of operational p*K' *values and correction for incomplete equilibration**. This supplement describes experimental determination of p*K'*_1 _and p*K'*_2 _from standard bicarbonate solutions (4, 8, and 16 mM NaHCO_3 _plus 50 mM NaCl). It also outlines the analytical procedure for the correction of incomplete equilibration of bicarbonate and hemolymph samples at low CO_2 _partial pressures. Click here for file

Additional file 4 **Reiterative least-squares spectral resolution & multicomponent analysis**. This supplement describes the *reiterative least-squares spectral resolution*, which was employed for the determination of the p*K'*_a _value and the acid/base spectra of cSNARF-1. It also outlines the *multicomponent analysis*, which was used to retrieve the *in vivo *pH from *in vivo *spectra of cSNARF. Click here for file
