# Supplementary material for: Physiological responses of Daphnia pulex to acid stress
Source: BMC Physiol. 2009 Apr 21;9:9. doi: 10.1186/1472-6793-9-9 (PMC2689847; doi:10.1186/1472-6793-9-9)
Supplement: Additional file 1 — Multiple sequence alignment of α-carbonic anhydrases. The α-CA sequences are divided into four groups according to similarity. Residues strictly conserved have a red background, residues well conserved within a group according to a Risler matrix [122] are indicated by red letters. Residues conserved between groups are boxed. Secondary structure elements of three human α-CAs are shown in blue on the top: helices with squiggles, beta strands with arrows, alpha and beta turns with TTT and TT letters. The numbering refers to HsCA2. Amino acid residues involved in zinc-binding and in the hydrogen-bonding network are indicated by red triangles. Yellow and orange backgrounds indicate mitochondrial targeting peptide or predicted signal peptides for secretory export. Pink and green backgrounds signify a transmembrane domain or potential glycosylphosphatidylinositol (GPI) anchor sites. Daphnia pulex sequences are indicated by red labels. Additionally included were related sequences from the blue crab Callinectes sapidus (Cs), Drosophila melanogaster (Dm), Anopheles gambiae (Ag), Caenorhabditis elegans (Ce), the sea urchin Strongylocentrotus purpuratus (Sp), and Homo sapiens (Hs). Sequences were aligned using the T-Coffee algorithm [158] and displayed with ESPript [120,161]. Sequence references, protein data bank (PDB) codes and NCBI accession numbers: Callinectes [124], Drosophila [119], Anopheles [125], HsCA2 (1CA2), HsCA4 (1ZNC), HsCA5A (NP_001730), HsCA6 (P23280), HsCA10 (AAH29865), HsCA12 (1JCZ), CeCAH2 (Q18932), SpCA8 (XP_795365), SpCAc (XP_782997), SpCA-RP (XP_784796), SpCA-GPI (XP_796525). [file 1472-6793-9-9-S1.pdf]

HsCA2

HsCA4
HsCA12

```
1 HsCA5A    ...MLGRNTWKTSAFSF.....L...........................................................................................................V
  SpCA8     ...MSILKFTKFRKRYL.....R...........................................................................................................G
  HsCA2     ...................................................................................................................................
  SpCAc     ...................................................................................................................................
  CsCAc     ...................................................................................................................................
  DmCG7820  ...................................................................................................................................
  AgCA9     ...................................................................................................................................
  CAA1      ...................................................................................................................................
  CAA2      ...MVWTV.....GDWCICVPES...........................................................................................................I
2 HsCA10    .MEIVWEVLFLLQANFIVC...............................................................................................................I
  SpCA-RP   ...................................................................................................................................
  CeCAH2    ..........MIPWLLTACI...............................................................................................................
  CAA3      ...MVDSVRFRLRTSCCISVSLA...........................................................................................................I
  AgCA-RP5  ....AHYHDLILA.FPLLF...............................................................................................................L
  DmCG32698 .......MELLQA.LCCTLLLLL...........................................................................................................A
  CAA4      .........MKIL.NCVVLYLFT............................................................................................................
  AgCA-RP2  .......IQTIHA.MCVLFVSVF............................................................................................................
  DmCG1402  ......PLDLIGI.FAHSLSLLL............................................................................................................
3 HsCA4     .....MRMLLAL..........L...........................................................................................................A
  HsCA6     .....MRALVLL..........L...........................................................................................................S
  HsCA12    ...MPRRSLHAAAVLLLVI...L...........................................................................................................K
  SpCA-GPI  ............MKFFV.....C...........................................................................................................V
  AgCA10    ....MKS..FTLL.ICY..................................................................................................................
  DmCG3940  ........MHLIALSLIVC................................................................................................................
  DmCG6906  MRRCRNTPFAIVIAPILIC................................................................................................................
  AgCA4     ......MMAATTT.TATTTTTTM..AARVGHCSR..............................................SHRRRGSSGAGGG................................VMLIAS
  CsCAg     ..........MVALQVVICV..S...........................................................................................................V
  CAA5      .....MPKEAVGV.FSILI...............................................................................................................L
4 CAA6H     ....MIFKISQLI.QIFVVSFLLFVE..................LVNGRPFAYHYNLI.....................................DSSHHEIDMNVKVESAPDDNI...............
  CAA6D     ....MMPQSSLTC.CPFVMFIVFVLLLI................SAAGFPVSDEAYPSS........................................................................
  CAA6C     .................................................................................................................MPWLNSFKSLTYYIIIIF
  CAA6G     ....MTEKLLLLK.YILAFLVCSSFLQ.................IVNNSPIRNVTPTK....................................GKNHRAVINPNIMVRTH....................
  CAA6E     ....MIVKNTQKK.KLFALTFLRVLETG..................................................................LSRRNHGQLSFQICVRGRY...................
  CAA6B     .....MPRPSVIL.IVFLVCFRFVTPSSTDDTLE..............................................ALPTTA.............................................
  CAA6F     ..MQVSRPNGILA.IVVVFMGLLAPTKCFPIIGEPFVAPEFLPTTARANPAIPEAIPYTRIPYTPSTAAPKENAAYPSPSTYSYTLKPEVTTTALSSTSDKIQSIVATTTSTIDAPGLAATSAITTSSIIPS
  CAA7F     ......MKTKLIS.ALMLVLLGFTR.............................................................................................RLMGAGILTSVVPT
  CAA7D     ....MLDKMFTLL.QIALVVSLG.....................LANGAVIR...............................................................................
  CAA7E     .....MILFQ....IVATIIGCVVVIKG................QDPANSVT...............................................................................
  CAA7C     .....MATSSLLF.KVLPALCCVVVINA..................QQTVVS...............................................................................
  CAA7G     .....MVKTNLLC.QLLLVMCCISLV..................VVSAMPQR..........................................SFKSDMKEITNTSL........................
  CAA7A     .....MAKLSVFS.QFFLVLCCFVAIN........................................................................................................
  CAA7I     .....MIKVALLFKFSL.................................................................................................................I
  CAA7M     .....MMKVASLFKFWL.....M...........................................................................................................I
  CAA7H     .....MSLDS..RKFLK.LLMCFAA..........................................................................................................
  CAA7P     .....MTMEATLLCKFL.LLDLLVN..........................................................................................................
  CAA7O     .....MTKAAPLLCKIA.LFGLLVN..........................................................................................................
  CAA7K     .....MSISS.LRKIAI.LAGLFVN..........................................................................................................
  CAA7J     .....MVKKASLACKLL.LLICYAA..........................................................................................................
  CAA7N     .....MVEKCSLACQVL.VLTCFSA..........................................................................................................
  CAA7Q     .....MVEKCSLTCQVY.MLSVNFF..........................................................................................................
```

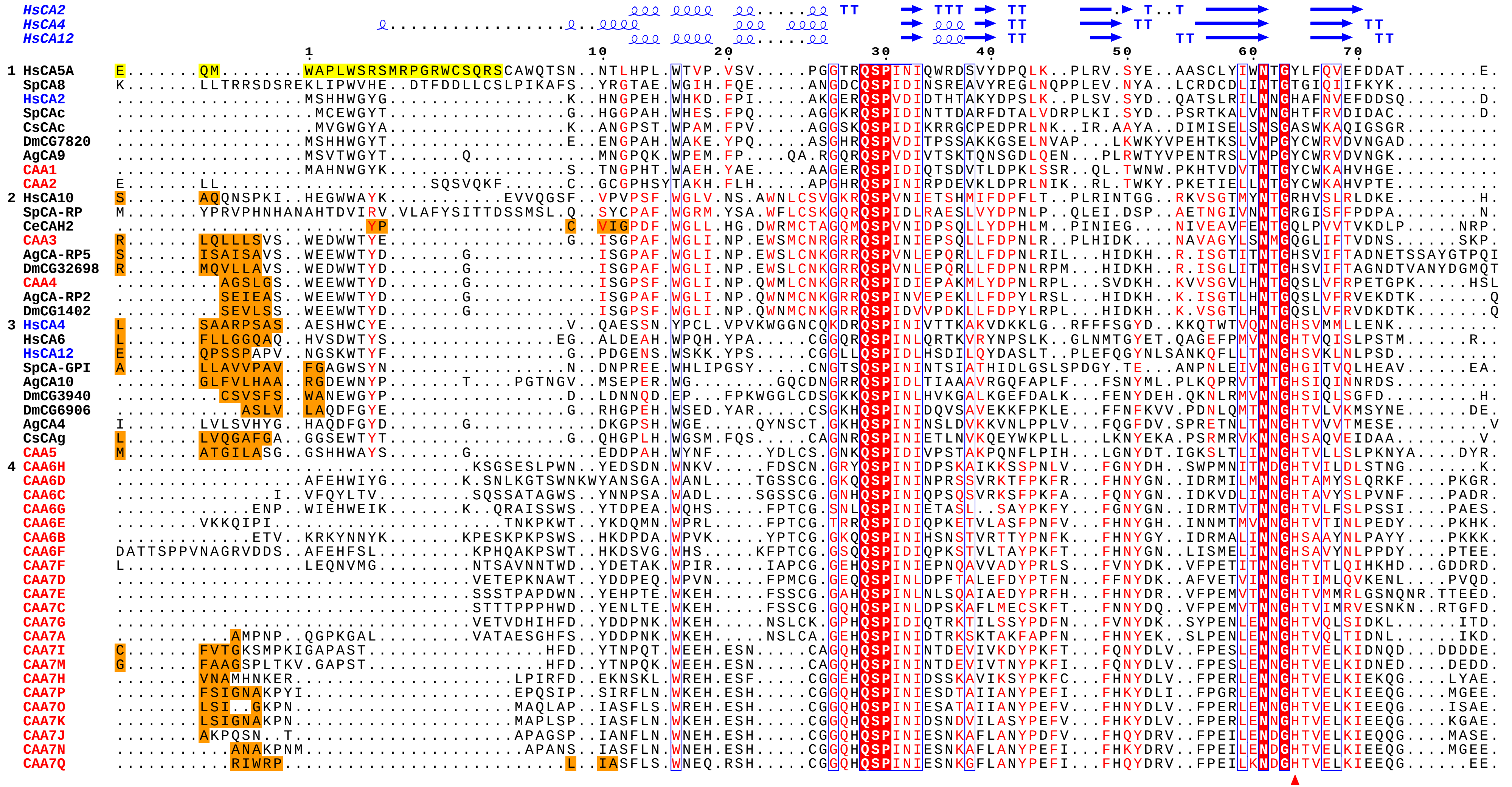
HsCA2
HsCA4
HsCA12
1 10 20 30 40 50 60 70
1 HsCA5A
SpCA8
HsCA2
SpCAc
CsCAc
DmCG7820
AgCA9
CAA1
CAA2
2 HsCA10
SpCA-RP
CeCAH2
CAA3
AgCA-RP5
DmCG32698
CAA4
AgCA-RP2
DmCG1402
3 HsCA4
HsCA6
HsCA12
SpCA-GPI
AgCA10
DmCG3940
DmCG6906
AgCA4
CsCAg
CAA5
4 CAA6H
CAA6D
CAA6C
CAA6G
CAA6E
CAA6B
CAA6F
CAA7F
CAA7D
CAA7E
CAA7C
CAA7G
CAA7A
CAA7I
CAA7M
CAA7H
CAA7P
CAA7O
CAA7K
CAA7J
CAA7N
CAA7Q

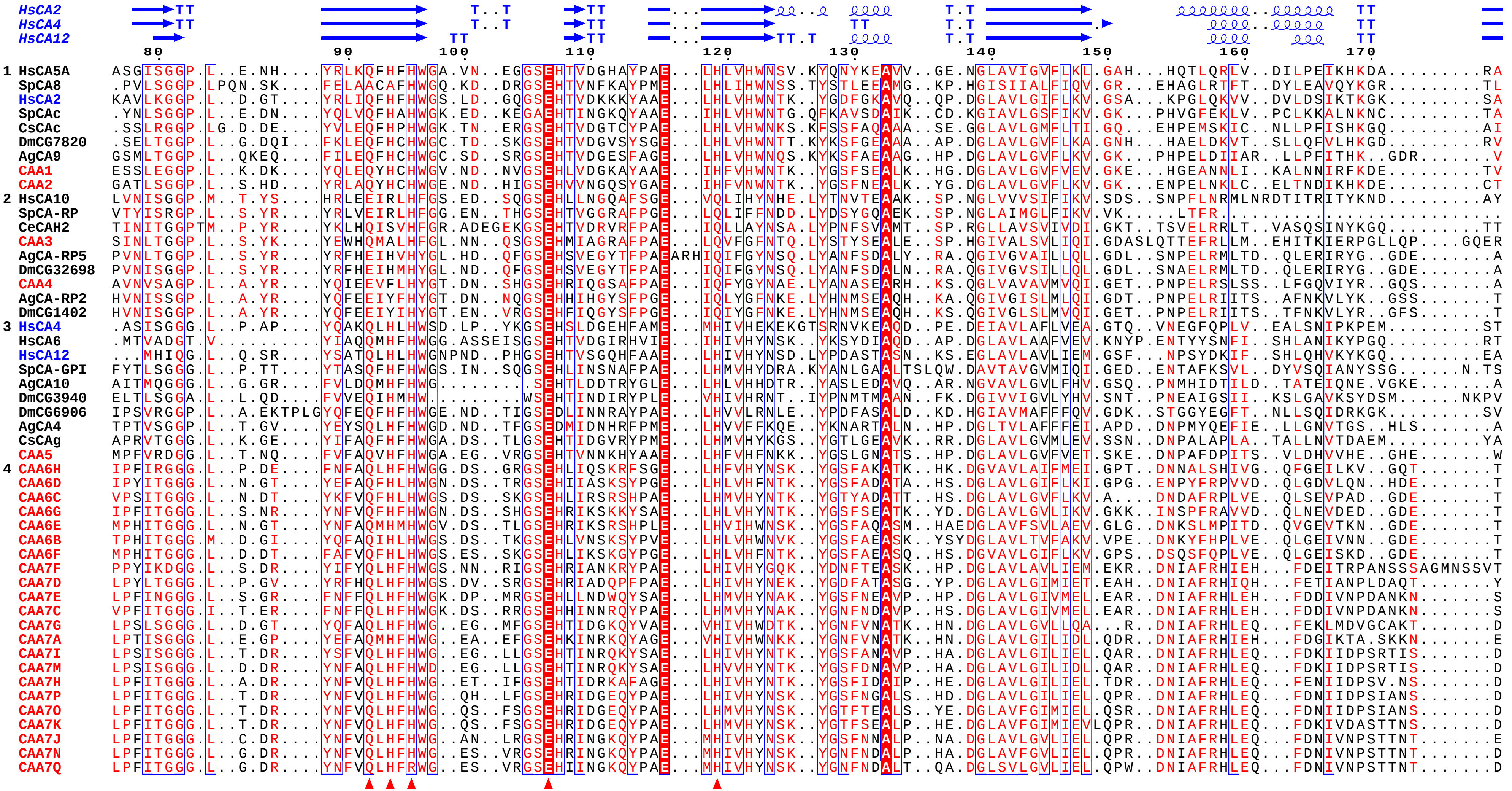
HsCA2
HsCA4
HsCA12
80 90 100 110 120 130 140 150 160 170
1 HsCA5A ASGISGGP.L..E.NH....YRLKQFHFHWGA.VN..EGGSEHTVDGHAYPAE...LHLVHWNSV.KYQNYKEAVV..GE.NGLAVIGVFLKL.GAH...HQTLQRLV..DILPEIKHKDA.........RA
SpCA8 .PVLSGGP.LPQN.SK....FELAACAFHWGQ.KD..DRGSEHTVNFKAYPME...LHLIHWNSS.TYSTLEEAMG..KP.HGISIIALFIQV.GR...EHAGLRTFT..DYLEAVQYKGR.........TL
HsCA2 KAVLKGGP.L..D.GT....YRLIQFHFHWGS.LD..GQGSEHTVDKKKYAAE...LHLVHWNTK..YGDFGKAVQ..QP.DGLAVLGIFLKV.GSA...KPGLQKVV..DVLDSIKTKGK.........SA
SpCAc .YNLSGGP.L..E.DN....YQLVQFHAHWGK.ED..KEGAEHTINGKQYAAE...IHLVHWNTG.QFKAVSDAIK..CD.KGIAVLGSFIKV.GK...PHVGFEKLV..PCLKKALNKNC.........TA
CsCAc .SSLRGGP.LG.D.DE....YVLEQFHPHWGK.TN..ERGSEHTVDGTCYPAE...LHLVHWNKS.KFSSFAQAAA..SE.GGLAVLGMFLTI.GQ...EHPEMSKIC..NLLPFISHKGQ.........AI
DmCG7820 .SELTGGP.L..G.DQI...FKLEQFHCHWGC.TD..SKGSEHTVDGVSYSGE...LHLVHWNTT.KYKSFGEAAA..AP.DGLAVLGVFLKA.GNH...HAELDKVT..SLLQFVLHKGD.........RV
AgCA9 GSMLTGGP.L...QKEQ...FILEQFHCHWGC.SD..SRGSEHTVDGESFAGE...LHLVHWNQS.KYKSFAEAAG..HP.DGLAVLGVFLKV.GK...PHPELDIIAR..LLPFITHK..GDR.......V
CAA1 ESSLEGGP.L..K.DK....YQLEQYHCHWGV.ND..NVGSEHLVDGKAYAAE...IHFVHWNTK..YGSFSEALK..HG.DGLAVLGVFVEV.GKE...HGEANNLI..KALNNIRFKDE.........TV
CAA2 GATLSGGP.L..S.HD....YRLAQYHCHWGE.ND..HIGSEHVVNGQSYGAE...IHFVNWNTK..YGSFNEALK..YG.DGLAVLGVFLKV.GK...ENPELNKLC..ELTNDIKHKDE.........CT
2 HsCA10 LVNISGGP.M..T.YS....HRLEEIRLHFGS.ED..SQGSEHLLNGQAFSGE...VQLIHYNHE.LYTNVTEAAK..SP.NGLVVVSIFIKV.SDS..SNPFLNRMLNRDTITRITYKND.........AY
SpCA-RP VTYISRGP.L..S.YR....YRLVEIRLHFGG.EN..THGSEHTVGGRAFPGE...LQLIFFNDD.LYDSYGQAEK..SP.NGLAIMGLFIKV.VK.....LTFR..................................
CeCAH2 TINITGGPTM..P.YR....YKLHQISVHFGR.ADEGEKGSEHTVDRVRFPAE...IQLLAYNSA.LYPNFSVAMT..SP.RGLLAVSVIVDI.GKT.TSVELRRLT..VASQSINYKGQ.........TT
CAA3 SINLTGGP.L..S.YK....YEWHQMALHFGL.NN..QSGSEHMIAGRAFPAE...LQVFGFNTQ.LYSTYSEALE..SP.HGIVALSVLIQI.GDASLQTTEFRLLM..EHITKIERPGLLQP....GQER
AgCA-RP5 PVNLTGGP.L..S.YR....YRFHEIHVHYGL.HD..QFGSEHSVEGYTFPAEARHIQIFGYNSQ.LYANFSDALY..RA.QGIVGVAILLQL.GDL..SNPELRMLTD..QLERIRYG..GDE.......A
DmCG32698 PVNISGGP.L..S.YR....YRFHEIHMHYGL.ND..QFGSEHSVEGYTFPAE...IQIFGYNSQ.LYANFSDALN..RA.QGIVGVSILLQL.GDL..SNAELRMLTD..QLERIRYG..GDE.......A
CAA4 AVNVSAGP.L..A.YR....YQIEEVFLHYGT.DN..SHGSEHRIQGSAFPAE...IQFYGYNAE.LYANASEARH..KS.QGLVAVAVMVQI.GET..PNPELRSLSS..LFGQVIYR..GQS.......A
AgCA-RP2 HVNISSGP.L..A.YR....YQFEEIYFHYGT.DN..NQGSEHHIHGYSFPGE...IQLYGFNKE.LYHNMSEAQH..KA.QGIVGISLMLQI.GDT..PNPELRIITS..AFNKVLYK..GSS.......T
DmCG1402 HVNISGGP.L..A.YR....YQFEEIYIHYGT.EN..VRGSEHFIQGYSFPGE...IQIYGFNKE.LYHNMSEAQH..KS.QGIVGLSLMVQI.GET..PNPELRIITS..TFNKVLYR..GFS.......T
3 HsCA4 .ASISGGG.L..P.AP....YQAKQLHLHWSD.LP..YKGSEHSLDGEHFAME...MHIVHEKEKGTSRNVKEAQD..PE.DEIAVLAFLVEA.GTQ..VNEGFQPLV..EALSNIPKPEM.........ST
HsCA6 .MTVADGT.V..........YIAQQMHFHWGG.ASSEISGSEHTVDGIRHVIE...IHIVHYNSK..YKSYDIAQD..AP.DGLAVLAAFVEV.KNYP.ENTYYSNFI..SHLANIKYPGQ.........RT
HsCA12 ...MHIQG.L..Q.SR....YSATQLHLHWGNPND..PHGSEHTVSGQHFAAE...LHIVHYNSD.LYPDASTASN..KS.EGLAVLAVLIEM.GSF...NPSYDKIF..SHLQHVKYKGQ.........EA
SpCA-GPI FYTLSGGG.L..P.TT....YTASQFHFHWGS.IN..SQGSEHLINSNAFPAE...LHMVHYDRA.KYANLGAALTSLQW.DAVTAVGVMIQI.GED..ENTAFKSVL..DYVSQIANYSSG.....N.TS
AgCA10 AITMQGGG.L..G.GR....FVLDQMHFHWG.........SEHTLDDTRYGLE...LHLVHHDTR..YASLEDAVQ..AR.NGVAVLGVLFHV.GSQ..PNMHIDTILD..TATEIQNE.VGKE......A
DmCG3940 ELTLSGGA.L..L.QD....FVVEQIHMHW.........WSEHTINDIRYPLE...VHIVHRNT..IYPNMTMAAN..FK.DGIVVIGVLYHV.SNT..PNEAIGSII..KSLGAVKSYDSM......NKPV
DmCG6906 IPSVRGGP.L..A.EKTPLGYQFEQFHFHWGE.ND..TIGSEDLINNRAYPAE...LHVVLRNLE..YPDFASALD..KD.HGIAVMAFFFQV.GDK..STGGYEGFT..NLLSQIDRKGK.........SV
AgCA4 TPTVSGGP.L..T.GV....YEYSQLHFHWGD.ND..TFGSEDMIDNHRFPME...LHVVFFKQE..YKNARTALN..HP.DGLTVLAFFFEI.APD..DNPMYQEFIE..LLGNVTGS..HLS.......A
CsCAg APRVTGGG.L..K.GE....YIFAQFHFHWGA.DS..TLGSEHTIDGVRYPME...LHMVHYKGS..YGTLGEAVK..RR.DGLAVLGVMLEV.SSN..DNPALAPLA..TALLNVTDAEM.........YA
CAA5 MPFVRDGG.L..T.NQ....FVFAQVHFHWGA.EG..VRGSEHTVNNKHYAAE...LHFVHFNKK..YGSLGNATS..HP.DGLAVLGVFVET.SKE..DNPAFDPITS..VLDHVVHE..GHE.......W
4 CAA6H IPFIRGGG.L..P.DE....FNFAQLHFHWGG.DS..GRGSEHLIQSKRFSGE...LHFVHYNSK..YGSFAKATK..HK.DGVAVLAIFMEI.GPT..DNNALSHIVG..QFGEILKV..GQT.......T
CAA6D IPYITGGG.L..N.GT....YEFAQFHLHWGN.DS..TRGSEHIIASKSYPGE...LHLVHFNTK..YGSFADATA..HS.DGLAVLGIFLKI.GPG..ENPYFRPVVD..QLGDVLQN..HDE.......T
CAA6C VPSITGGG.L..N.DT....YKFVQFHLHWGS.DS..SKGSEHLIRSRSHPAE...LHMVHYNTK..YGTYADATT..HS.DGLAVLGVFLKV.A....DNDAFRPLVE..QLSEVPAD..GDE.......T
CAA6G IPFITGGG.L..S.NR....YNFVQFHFHWGN.DS..SHGSEHRIKSKKYSAE...LHLVHYNTK..YGSFSEATK..YD.DGLAVLGVLIKV.GKK..INSPFRAVVD..QLNEVDED..GDE.......T
CAA6E MPHITGGG.L..N.GT....YNFAQMHMHWGV.DS..TLGSEHRIKSRSHPLE...LHVIHWNSK..YGSFAQASM..HAEDGLAVFSVLAEV.GLG..DNKSLMPITD..QVGEVTKN..GDE.......T
CAA6B TPHITGGG.M..D.GI....YQFAQIHLHWGS.DS..TKGSEHLVNSKSYPVE...LHLVHWNVK..YGSFAEASK..YSYDGLAVLTVFAKV.VPE..DNKYFHPLVE..QLGEIVNN..GDE.......T
CAA6F MPHITGGG.L..D.DT....FAFVQFHLHWGS.ES..SKGSEHLIKSKGYPGE...LHLVHFNTK..YGSFAEASQ..HS.DGVAVLGIFLKV.GPS..DSQSFQPLVE..QLGEISKD..GDE.......T
CAA7F PPYIKDGG.L..S.DR....YIFYQLHFHWGS.NN..RIGSEHRIANKRYPAE...LHIVHYGQK..YDNFTEASK..HP.DGLAVLAVLIEM.EKR..DNIAFRHIEH...FDEITRPANSSSAGMNSSVT
CAA7D LPYLTGGG.L..P.GV....YRFHQLHFHWGS.DV..SRGSEHRIADQPFPAE...LHIVHYNEK..YGDFATASG..YP.DGLAVLGIMIET.EAH..DNIAFRHIQH...FETIANPLDAQT.......Y
CAA7E LPFINGGG.L..S.GR....FNFFQLHFHWGK.DP..MRGSEHLLNDWQYSAE...LHMVHYNAK..YGSFNEAVP..HP.DGLAVLGIVMEL.EAR..DNIAFRHLEH...FDDIVNPDANKN.......S
CAA7C VPFITGGG.I..T.ER....FNFFQLHFHWGK.DS..RRGSEHHINNRQYPAE...LHMVHYNAK..YGNFNDAVP..HS.DGLAVLGIVMEL.EAR..DNIAFRHLEH...FDDIVNPDANKN.......S
CAA7G LPSLSGGG.L..D.GT....YQFAQLHFHWG..EG..MFGSEHTIDGKQYVAE...VHIVHWDTK..YGNFVNATK..HN.DGLAVLGVLLQA...R..DNIAFRHLEQ...FEKLMDVGCAKT.......D
CAA7A LPTISGGG.L..E.GP....YEFAQMHFHWG..EA..EFGSEHKINRKQYAGE...VHIVHWNKK..YGNFVNATK..HN.DGLAVLGILIDL.QDR..DNIAFRHIEH...FDGIKTA.SKKN.......E
CAA7I LPSISGGG.L..T.DR....YSFVQLHFHWG..EG..LLGSEHTINRQKYSAE...LHIVHYNTK..YGSFANAVP..HA.DGLAVLGILIEL.QAR..DNIAFRHLEQ...FDKIIDPSRTIS.......D
CAA7M LPSISGGG.L..D.DR....YNFAQLHFHWD..EG..LLGSEHTINRQKYSAE...LHVVHYNTK..YGSFDNAVP..HA.DGLAVLGILIDL.QAR..DNIAFRHLEQ...FDKIIDPSRTIS.......D
CAA7H LPFITGGG.L..A.DR....YNFVQLHFHWG..ET..IFGSEHTIDRKAFAGE...LHIVHYNTK..YGSFIDAIP..HE.DGLAVLGILIEL.TDR..DNIAFRHLEQ...FENIIDPSV.NS.......D
CAA7P LPFITGGG.L..T.DR....YNFVQLHFHWG..QH..LFGSEHRIDGEQYPAE...LHIVHYNSK..YGSFNGALS..HD.DGLAVLGILIEL.QPR..DNIAFRHLEQ...FDNIIDPSIANS.......D
CAA7O LPFITGGG.L..T.DR....YNFVQLHFHWG..QS..FSGSEHRIDGEQYPAE...LHMVHYNSK..YGTFTEALS..YE.DGLAVFGIMIEL.QSR..DNIAFRHLEQ...FDNIIDPSIANS.......D
CAA7K LPFITGGG.L..T.DR....YNFVQLHFHWG..QS..FSGSEHRIDGEQYPAE...LHMVHYNSK..YGTFSEALP..HE.DGLAVFGIMIEVLQPR..DNIAFRHLEQ...FDKIVDASTTNS.......D
CAA7J LPFITGGG.L..C.DR....YNFVQLHFHWG..AN..LRGSEHRINGKQYPAE...MHIVHYNSK..YGSFNNALP..NA.DGLAVLGVLIEL.QPR..DNIAFRHLEQ...FDNIVNPSTTNT.......E
CAA7N LPFITGGG.L..G.DR....YNFVQLHFHWG..ES..VRGSEHRINGKQYPAE...MHIVHYNSK..YGNFNDALP..HA.DGLAVFGVLIEL.QPR..DNIAFRHLEQ...FDNIVNPSTTNT.......D
CAA7Q LPFITGGG.L..G.DR....YNFVQLHFRWG..ES..VRGSEHIINGKQYPAE...MHIVHYNSK..YGNFNDALT..QA.DGLSVLGVLIEL.QPW..DNIAFRHLEQ...FDNIVNPSTTNT.......D

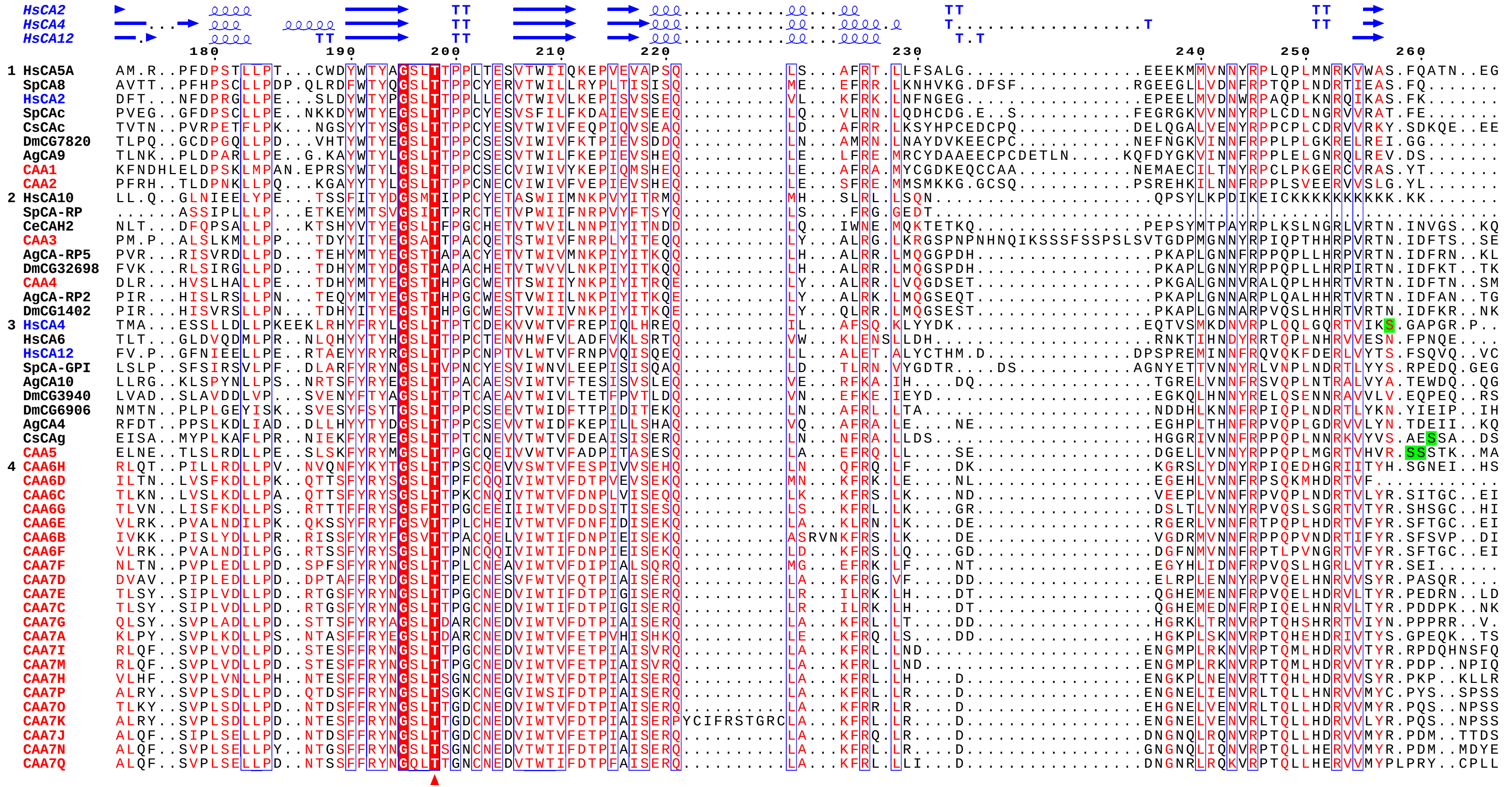
HsCA2
HsCA4
HsCA12
180
190
200
210
220
230
240
250
260
1 HsCA5A
SpCA8
HsCA2
SpCAc
CsCAc
DmCG7820
AgCA9
CAA1
CAA2
2 HsCA10
SpCA-RP
CeCAH2
CAA3
AgCA-RP5
DmCG32698
CAA4
AgCA-RP2
DmCG1402
3 HsCA4
HsCA6
HsCA12
SpCA-GPI
AgCA10
DmCG3940
DmCG6906
AgCA4
CsCAg
CAA5
4 CAA6H
CAA6D
CAA6C
CAA6G
CAA6E
CAA6B
CAA6F
CAA7F
CAA7D
CAA7E
CAA7C
CAA7G
CAA7A
CAA7I
CAA7M
CAA7H
CAA7P
CAA7O
CAA7K
CAA7J
CAA7N
CAA7Q

HsCA2
HsCA4
HsCA12

```
1 HsCA5A     TRS.......................................................................................................................................
  SpCA8      ..................................................................................................................................
  HsCA2      ..................................................................................................................................
  SpCAc      .................................................................................................................................N
  CsCAc      ..................................................................................................................................
  DmCG7820   .................................................................................................................................H
  AgCA9      .................................................................................................................................Y
  CAA1       ..................................................................................................................................
  CAA2       ..................................................................................................................................
2 HsCA10     ..................................................................................................................................
  SpCA-RP    ..................................................................................................................................
  CeCAH2     STV..........................................SSSCPSNVYYVEMGYQANPGRNKRNDSVSRRYVPTSEVFEIDSIRPDDV..........SKA....................GSF
  CAA3       RG...........................................EK.ACATMHRDMHYRGEFS.......FEIAKTFFAVFFSSRTH..................S....................VHK
  AgCA-RP5   E...........................................KGGKICATMYKDVHYKGRR..................GWVMWGK..................K...................ENIQ
  DmCG32698  S...........................................NGKAACPTMYREVYYKATS......................W.........................................KQN
  CAA4       E...........................................RG...CVTMKTDMSYKANS......................W....................N...................IATT
  AgCA-RP2   V...........................................SKTQSCPTMYKDMYYKANR......................W.........................................TSE
  DmCG1402   V...........................................SGIALINPLAATRLPKWTR......................FLF..................V...................VTSL
3 HsCA4      .............................................................LPWALPAL..LGPMLACLLAGFL.............................................R
  HsCA6      .............................................................YTLGSEFQ..FYLHKIEEILDYLRRAL.........................................N
  HsCA12     TAA............................................GLSLGIILS.....LALAGILG..ICIVVVVSIWLFRRKSIKKGDNKGVIYKPATKMETE....................AHA
  SpCA-GPI   DET............................................ST.....PE.....PTDGGAVA..ISPMRALFIVALALCML..................F....................NRF
  AgCA10     N..............................................NFATKMTSNVVFLGAI..................VLLVITS..................R...................LSYH
  DmCG3940   GSA............................................GLTASVS....................LGLMTLILAGQKFL.........................................L
  DmCG6906   NMG............................................SIPLVDAEN...AAGKWRAQAA..AVLLPLVVLAALSRTSIF......................................RGF
  AgCA4      VDLDGEQPEEPSEAGVDTNQLDTGRSDEAPKAPSDPNGRKHPRNAGTPSIATRWSSVLVVALP..................LLWAAFL..................R...................SERY
  CsCAg      STM............................................KK...........................................VGLTFYFMTSMAVLM....................MNM
  CAA5       A...........................................SLVAFLLPIVA.......................................................................VLAL
4 CAA6H      E..........PLQSWEQN....ILDFFKSSQFDS...........EQFFKWRPFSHFDKLTN..................PL....E..................I...................PNWS
  CAA6D      ..................................................................................................................................
  CAA6C      P..........RSWP...ISWTPLLDAIKWSQCIIG..............................................FTLFR..........................................Q
  CAA6G      T..........INWS...HISSPIVDIYQYSTCVFSFYANSFLT...........................................I..................L...................GVNL
  CAA6E      S..........RSLS....DATSMADVFKWTKCLMS..............................................YAFNSI..................F...................S.SS
  CAA6B      P..........LSHPPSVNSYSPFSEAYKWSKATFKNYATRFLG.............IFDHPY..................PHRYNSC..................R...................SRPC
  CAA6F      P..........RSWP...ITSSPFSEAYKWSKCLMG..............................................FAYKSV..................A...................PSSL
  CAA7F      ..................................................................................................................................
  CAA7D      .................................................................................................................................L
  CAA7E      E.................................................................................................................................
  CAA7C      N.................................................................................................................................
  CAA7G      .................................................................................................................................A
  CAA7A      E.............................................LPLVFPIYNPAAFAYYP..................FFPQNVP..................F...................MMVY
  CAA7I      PLF............................................PL............................................FPDYSNLFKREFQP...................SFNF
  CAA7M      SLF............................................SFPSN.........................................LPNLFKPFSNEFQQ...................S.NF
  CAA7H      PLF............................................LLPN.............................................IFTSFPNNWSI.................IDITSL
  CAA7P      PLL............................................PFSDTE...................................................FSP.................MEFPLN
  CAA7O      PSL............................................SNPSSPL...LSLP..................................DLIS.......A.................IEFPLE
  CAA7K      PLL............................................SNPSNPLSPLLSNPLSPLLSSNPS........................SPLLSLPFLIPD.................IEIAFE
  CAA7J      QPN............................................FNPSNP...FFAFP..................................NIFSAFPNVFQS......................N
  CAA7N      PNF.............................................NPTNP...FLSFP..................................NIFSAFPNVFQSFQSNYERN..............Y
  CAA7Q      PRY...................................DRFIRTEFQSNKSIC...LYS....................................QRFQMYFNQIKQSCKSVDCTVLTPIRLSAPQLLFL
```
