# Supplementary material for: Physiological responses of Daphnia pulex to acid stress
Source: BMC Physiol. 2009 Apr 21;9:9. doi: 10.1186/1472-6793-9-9 (PMC2689847; doi:10.1186/1472-6793-9-9)
Supplement: Additional file 2 — Multiple sequence alignment of β-carbonic anhydrases. Numbering and the secondary structure elements on the top refer to the β-CA from Pisum sativum (PsCAb) [121]. The other sequences are from Daphnia pulex (CAB), Drosophila melanogaster (DmCG11967), Anopheles gambiae (AgCAb), Caenorhabditis elegans (CeCAb1), sea urchin Strongylocentrotus purpuratus (SpCAb), and the sea anemone Nematostella vectensis (NvCAb). A column is framed in blue if more than 70% of its residues are similar according to physico-chemical properties. Similar residues are indicated by red letters; strictly conserved residues have a red background. Secondary structure elements are presented as follows: helices with squiggles, beta strands with arrows, alpha and beta turns with TTT and TT letters. Amino acid residues involved in zinc and substrate binding are indicated by red and blue triangles. Sequences were aligned using the T-Coffee algorithm [158] and displayed with ESPript [120,161]. Protein data bank (PDB) code and NCBI accession numbers: PsCAb (2EKJ), DmCG11967 (NP_649849), AgCAb (XP_563117), CeCAb1 (NP_741809), SpCAb (XP_786120), NvCAb (XP_001632619). [file 1472-6793-9-9-S2.pdf]

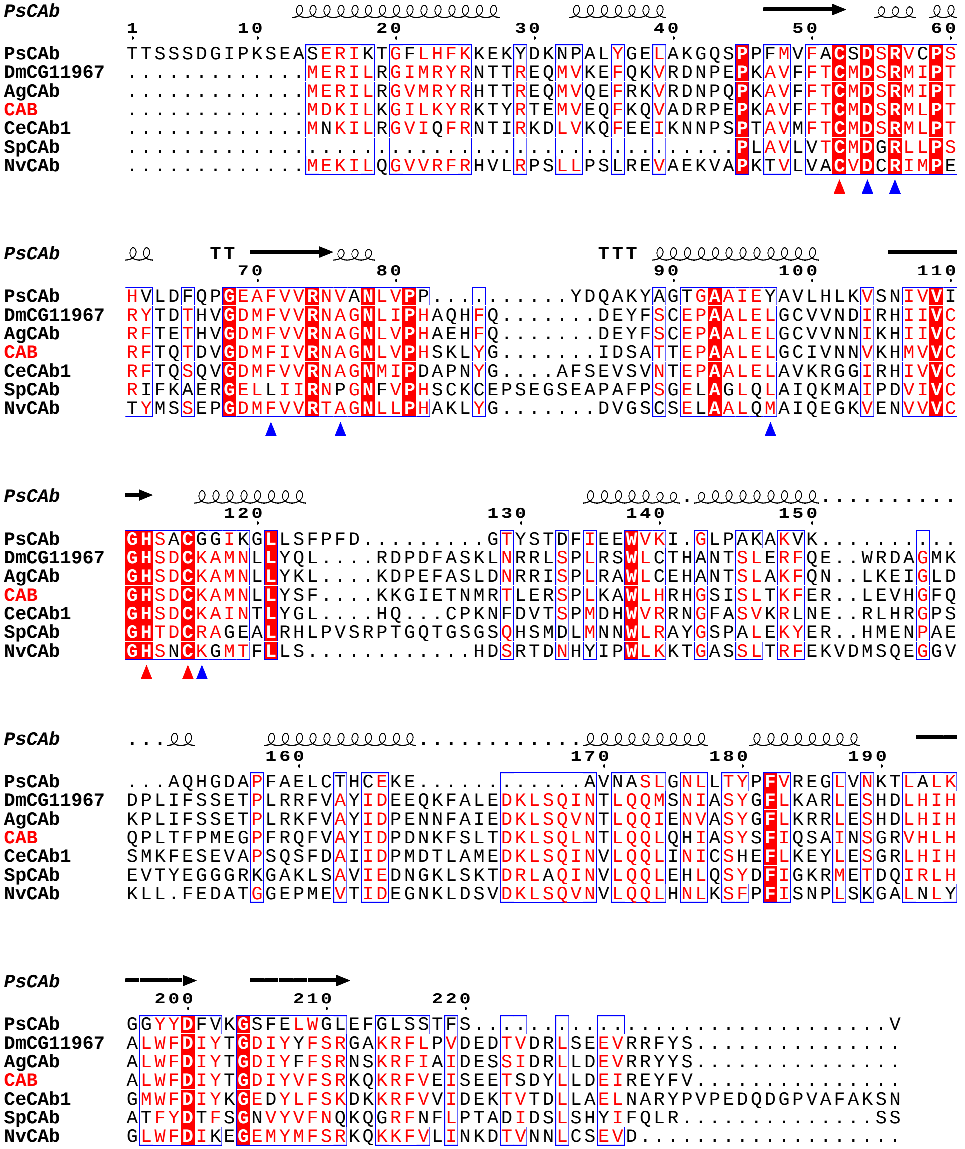
PsCAb
1 10 20 30 40 50 60
PsCAb TTSSSDGIPKSEASERIKTGFLHFKKEKYDKNPALYGELAKGQSPPFMVFACSDSRVCPS
DmCG11967 .............MERILRGIMRYRNTTREQMVKEFQKVRDNPEPKAVFFTCMDSRMIPT
AgCAb .............MERILRGVMRYRHTTREQMVQEFRKVRDNPQPKAVFFTCMDSRMIPT
CAB .............MDKILKGILKYRKTYRTEMVEQFKQVADRPEPKAVFFTCMDSRMLPT
CeCAb1 .............MNKILRGVIQFRNTIRKDLVKQFEEIKNNPSPTAVMFTCMDSRMLPT
SpCAb ....PLAVLVTCMDGRLLPS
NvCAb .............MEKILQGVVRFRHVLRPSLLPSLREVAEKVAPKTVLVACVDCRIMPE
PsCAb
TT TTT
70 80 90 100 110
PsCAb HVLDFQPGEAFVVRNVANLVPP..........YDQAKYAGTGAAIEYAVLHLKVSNIVVI
DmCG11967 RYTDTHVGDMFVVRNAGNLIPHAQHFQ.......DEYFSCEPAALELGCVVNDIRHIIVC
AgCAb RFTETHVGDMFVVRNAGNLVPHAEHFQ.......DEYFSCEPAALELGCVVNNIKHIIVC
CAB RFTQTDVGDMFIVRNAGNLVPHSKLYG.......IDSATTEPAALELGCIVNNVKHMVVC
CeCAb1 RFTQSQVGDMFVVRNAGNMIPDAPNYG....AFSEVSVNTEPAALELAVKRGGIRHIVVC
SpCAb RIFKAERGELLIIRNPGNFVPHSCKCEPSEGSEAPAFPSGELAGLQLAIQKMAIPDVIVC
NvCAb TYMSSEPGDMFVVRTAGNLLPHAKLYG.......DVGSCSELAALQMAIQEGKVENVVVC
PsCAb
120 130 140 150
PsCAb GHSACGGIKGLLSFPFD..........GTYSTDFIEEWVKI.GLPAKAKVK.........
DmCG11967 GHSDCKAMNLLYQL....RDPDFASKLNRRLSPLRSWLCTHANTSLERFQE..WRDAGMK
AgCAb GHSDCKAMNLLYKL....KDPEFASLDNRRISPLRAWLCEHANTSLAKFQN..LKEIGLD
CAB GHSDCKAMNLLYSF....KKGIETNMRTLERSPLKAWLHRHGSISLTKFER..LEVHGFQ
CeCAb1 GHSDCKAINTLYGL....HQ...CPKNFDVTSPMDHWVRRNGFASVKRLNE..RLHRGPS
SpCAb GHTDCRAGEALRHLPVSRPTGQTGSGSQHSMDLMNNWLRAYGSPALEKYER..HMENPAE
NvCAb GHSNCKGMTFLLS............HDSRTDNHYIPWLKKTGASSLTRFEKVDMSQEGGV
PsCAb
160 170 180 190
PsCAb ...AQHGDAPFAELCTHCEKE............AVNASLGNLLTYPFVREGLVNKTLALK
DmCG11967 DPLIFSSETPLRRFVAYIDEEQKFALEDKLSQINTLQQMSNIASYGFLKARLESHDLHIH
AgCAb KPLIFSSETPLRKFVAYIDPENNFAIEDKLSQVNTLQQIENVASYGFLKRRLESHDLHIH
CAB QPLTFPMEGPFRQFVAYIDPDNKFSLTDKLSQLNTLQQLQHIASYSFIQSAINSGRVHLH
CeCAb1 SMKFESEVAPSQSFDAIIDPMDTLAMEDKLSQINVLQQLINICSHEFLKEYLESGRLHIH
SpCAb EVTYEGGGRKGAKLSAVIEDNGKLSKTDRLAQINVLQQLEHLQSYDFIGKRMETDQIRLH
NvCAb KLL.FEDATGGEPMEVTIDEGNKLDSVDKLSQVNVLQQLHNLKSFPFISNPLSKGALNLY
PsCAb
200 210 220
PsCAb GGYYDFVKGSFELWGLEFGLSSTFS..............................V
DmCG11967 ALWFDIYTGDIYYFSRGAKRFLPVDEDTVDRLSEEVRRFYS...............
AgCAb ALWFDIYTGDIYFFSRNSKRFIAIDESSIDRLLDEVRRYYS...............
CAB ALWFDIYTGDIYVFSRKQKRFVEISEETSDYLLDEIREYFV...............
CeCAb1 GMWFDIYKGEDYLFSKDKKRFVVIDEKTVTDLLAELNARYPVPEDQDGPVAFAKSN
SpCAb ATFYDTFSGNVYYVFNQKQGRFNFLPTADIDSLSHYIFQLR.............SS
NvCAb GLWFDIKEGEMYMFSRKQKKFVLINKDTVNNLCSEVD...................
