# Supplementary material for: Physiological responses of Daphnia pulex to acid stress
Source: BMC Physiol. 2009 Apr 21;9:9. doi: 10.1186/1472-6793-9-9 (PMC2689847; doi:10.1186/1472-6793-9-9)
Supplement: Additional file 3 — Determination of operational pK' values and correction for incomplete equilibration. This supplement describes experimental determination of pK'1 and pK'2 from standard bicarbonate solutions (4, 8, and 16 mM NaHCO3 plus 50 mM NaCl). It also outlines the analytical procedure for the correction of incomplete equilibration of bicarbonate and hemolymph samples at low CO2 partial pressures. [file 1472-6793-9-9-S3.pdf]

Supplement 3

Determination of operational *pK′* values of the carbonate system

The apparent pK-values of the carbonate system were experimentally determined from standard $NaHCO_3$ solutions (4, 8, and 16 mM) which additionally contained 50 mM NaCl. These standard solutions were equilibrated with gas mixtures of different CO_2 partial pressure (P_{CO2}) at 20 °C while the pH was continuously measured (Figure S1). The dependence of pH on P_{CO2} in a system containing bicarbonate and carbonate buffers is described by the following balance equation [1]

$$0 = \alpha_{CO_2} P_{CO_2} \left(\frac{K'_1}{\{H^+\}} + \frac{2K'_2}{\{H^+\}^2} \right) + \frac{K'_w}{\{H^+\}} - \{H^+\} - SID\,. \qquad (I)$$

where $\{H^+\}$ is 10^{-pH}, K'_w (= 10^{-14} M) is the dissociation constant of water, and SID (= $[Na^+] - [Cl^-]$) is the strong ion difference. The physical solubility of CO_2 (α_{CO2}) in water was assumed to be that of an aqueous solution containing 58 mM Na^+. For such a solution, an α_{CO2} value of 0.3682 mmol L^{-1} kPa^{-1} was calculated using a thermodynamic model [2,3], assuming a solution density of 1 g L^{-1}. Given the experimental data (pH *vs.* P_{CO2}) of the three standard solutions, best-fit parameter values for pK'_1 and pK'_2 (Figure S1) were obtained by implicitly solving Eq. (I) using the 'lsqnonlin' function from the optimization toolbox of Matlab 7.0 (MathWorks, Inc.).

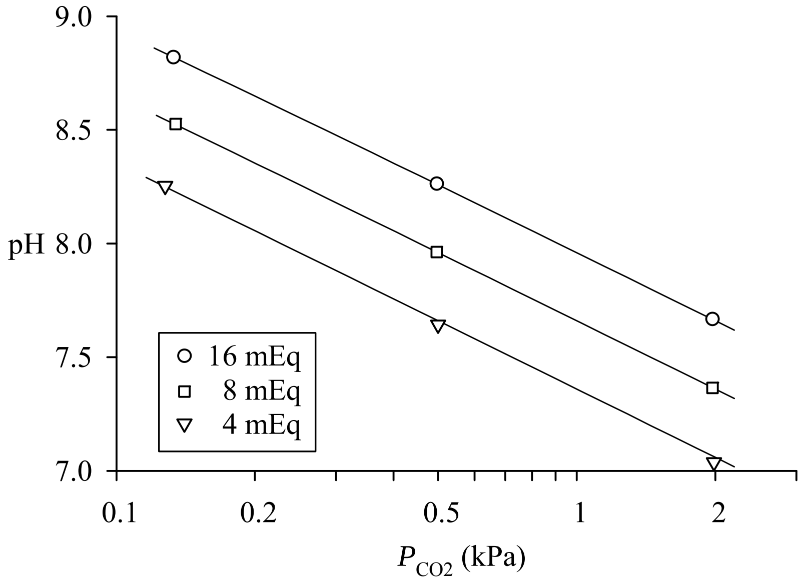

Figure S1 – Determination of operational p*K′* values for the carbonate system. Symbols show the measured dependence of pH on P_{CO2} for three standard $NaHCO_3$/NaCl solutions with strong ion differences (SID) of 4, 8, and 16 meq L^{-1}. Global data fitting (solid lines) using Eq. (I) yielded the best-fit parameter values of 6.325 ± 0.002 and 10.47 ± 0.09 (mean ± S.E.) for pK'_1 and pK'_2, respectively.

Correction for incomplete equilibration

At very low CO_2 partial pressures (0.12–0.14 kPa), it was not possible to completely equilibrate the bicarbonate and hemolymph samples within reasonable time. Based on the time course in pH when approaching equilibrium (Figure S2: inset) and the appropriate balance equation (e.g. Eq. I), the sample P_{CO2} prevailing at the end of the equilibration step was predicted by assuming that the average partial pressure in the liquid phase, $P_{CO2(aq)}$, approached the gas-phase $P_{CO2(g)}$ according to an exponential decay (Figure IIA: inset)

$$P_{CO_2(aq)}(t) = P_{CO_2(g)} + (a + P_{CO_2(g)})\exp(-bt) \tag{II}$$

where t is the time (min). The fitted parameters comprised the time constant b (min^{-1}) and the initial partial pressure a (kPa) in the sample at some initial time point.

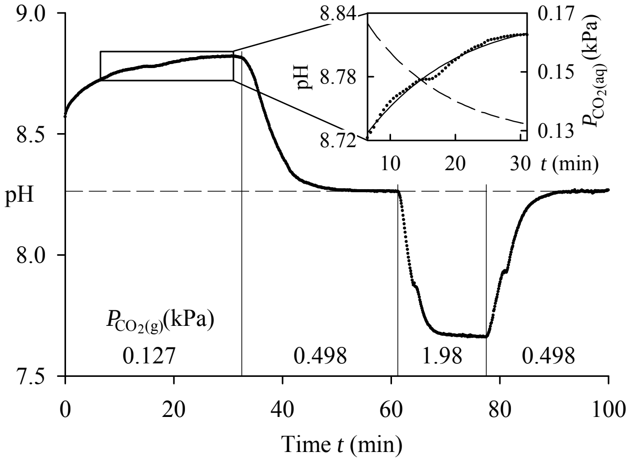

Figure S2 – Correction for incomplete equilibration. Shown is a pH profile from a 10 µl sample containing 16 mM $NaHCO_3$ and 50 mM NaCl during equilibration with gas mixtures of different CO_2 partial pressure ($P_{CO2(g)}$) at 20 °C. Note the good reproducibility (dashed line) of the measured pH at 0.498 kPa. Inset: Enlarged view of the pH profile illustrating the incomplete equilibration of the sample during the first incucation step at the lowest $P_{CO2(g)}$. Based on the time course of the measured pH (dotted line), the buffer model in Eq. (I) and the fitted p*K'* values, the average partial pressure in the liquid phase ($P_{CO2(aq)}$; dashed curve) was predicted by assuming that the $P_{CO2(aq)}$ approaches gas-phase $P_{CO2(g)}$ according to an exponential decay (Eq. II). The pH (solid line) calculated from the predicted $P_{CO2(aq)}$ shows an excellent agreement with the measured pH.

References

1. Truchot JP: **Comparative aspects of extracellular acid-base balance**. Berlin: Springer-Verlag; 1987.

2. Duan Z, Sun R: **An improved model calculating CO_2 solubility in pure water and aqueous NaCl solutions from 273 to 533 K and from 0 to 2000 bar**. *Chem Geol* 2003, **193(3-4):**257-271.

3. Duan Z, Sun R, Zhu C, Chou I-M: **An improved model for the calculation of CO_2 solubility in aqueous solutions containing Na^+, K^+, Ca^{2+}, Mg^{2+}, Cl^-, and SO_4^{2-}**. *Mar Chem* 2006, **98(2-4):**131-139.
