# Supplementary material for: Physiological responses of Daphnia pulex to acid stress
Source: BMC Physiol. 2009 Apr 21;9:9. doi: 10.1186/1472-6793-9-9 (PMC2689847; doi:10.1186/1472-6793-9-9)
Supplement: Additional file 4 — Reiterative least-squares spectral resolution & multicomponent analysis. This supplement describes the reiterative least-squares spectral resolution, which was employed for the determination of the pK'a value and the acid/base spectra of cSNARF-1. It also outlines the multicomponent analysis, which was used to retrieve the in vivo pH from in vivo spectra of cSNARF. [file 1472-6793-9-9-S4.pdf]

Supplement 4

Reiterative least-squares spectral resolution

The CO_2 titration of a bicarbonate-buffered, NaCl-containing solution of cSNARF-1 was not sufficient to shift the dye in the fully protonated (acid) and deprotonated (base) forms, whose spectra are necessary for pK'_a determination. *Reiterative least-squares spectral resolution* [1] was used to recover both the pK'_a value and the 'parent' spectra of the acid/base forms. In theory, each measured cSNARF-1 spectrum can be expressed as the sum of the two parent spectra, which are weighted according to the fractional content of the acid and base forms. The fraction (α) of acid at a particular pH is given by [1]

$$\alpha = 1\Big/\left(1+10^{\mathrm{pH}-\mathrm{p}K'_a}\right). \qquad \text{(I)}$$

The fraction of the conjugated base is consequently $\beta = 1 - \alpha$. A measured fluorescence spectrum can be represented as a column vector in which each element contains the emission intensity at a particular wavelength [2]. For each new spectrum at a different pH, another column vector is acquired. All column vectors are combined into a matrix **D**, where the wavelength of the measurement is the index for the rows and the pH is the index for the columns. This matrix **D** can be expressed as [1]

$$\mathbf{D} = \mathbf{AC} \qquad \text{(II)}$$

where **A** is a two-column matrix, whose first and second columns contain the difference (base minus acid) spectrum and sum spectrum, respectively, of the parent spectra. **C** is a two-row matrix, whose first row contains the difference distribution curve ($\beta - \alpha$) in dependence of pH, whereas the second row is all ones. Given the measurement of the data matrix **D** and the acid-base equilibrium model (equation I) for the distribution matrix **C**, all what is required to specify the spectral matrix **A** is the pK'_a value. Owing to the presence of noise in the experimental data, an exact solution for **A** in equation (II) is unlikely. However, for a given choice of pK'_a, the best spectral matrix $\hat{\mathbf{A}}$ can found by multiplying the data matrix **D** by the Moore-Penrose pseudoinverse of **C** ($\mathbf{C}^+$) [1]

$$\hat{\mathbf{A}} = \mathbf{DC}^+, \qquad \text{(III)}$$

which minimizes the sum of the squared residuals (SSR) between measured data (**D**) and the predicted data ($\hat{\mathbf{D}}$). The latter is obtained from

$$\hat{\mathbf{D}} = \hat{\mathbf{A}}\mathbf{C}. \qquad \text{(IV)}$$

The optimum pK'_a value yielding the best fit of the measured data was finally determined by minimizing the SSR with the Nelder-Mead simplex algorithm [3]. This optimization of pK'_a was additionally linked to (i) the simultaneous correction of each measured spectrum in **D** by an

additive offset factor to account for variations in CCD dark current and to (ii) the adjustment of each row in **C** by a multiplicative scaling factor to account for variations in excitation light intensity, sample shape, and fluorophore concentration.

Multicomponent analysis

In vivo cSNARF-1 fluorescence spectra were subjected to a *multicomponent analysis* to retrieve the *in vivo* pH. This method determines the composition of a mixture of components, given that the spectrum of each (pure) parent species is known [4]. The two parent (acid/base) spectra of cSNARF-1 were stored as column vectors in a four-column matrix **A**, which additionally contained a reference spectrum from non-injected animals and a constant offset spectrum. Given the measurement of the data matrix **D**, which contains the *in vivo* fluorescence spectra as column vectors, and the pseudoinverse of **A** ($\mathbf{A}^+$), the following expression [4]

$$\mathbf{C} = \mathbf{A}^+\mathbf{D} \qquad \text{(V)}$$

yields to the four-row matrix **C**, whose first and second rows contain the best-fit concentrations of the base and acid forms of cSNARF-1 in relation to the measurement number. The last two rows of **C** carry the best-fit contributions of the reference and offset spectra. From the concentrations of the acid/base forms, the fraction (α) of acid and the total concentration of cSNARF-1 can easily be derived.

References

1. Frans SD, Harris JM: **Reiterative least-squares spectral resolution of organic/acid base mixtures**. *Anal Chem* 1984, **56**(3):466-470.

2. Hendler RW, Shrager RI: **Deconvolutions based on singular value decomposition and the pseudoinverse: a guide for beginners**. *J Biochem Biophys Methods* 1994, **28**(1):1-33.

3. Press WH, Teukolsky SA, Vetterling WT, Flannery BP: **Numerical recipes in C: The art of scientific computing**, 2nd edn. Cambridge: Cambridge University Press; 1992.

4. Vandegriff KD, Shrager RI: **Hemoglobin-oxygen equilibrium binding: Rapid-scanning spectrophotometry and singular value decomposition**. In: *Methods in Enzymology, vol 232, Hemoglobins Part C: Biophysical methods.* Edited by Everse J, Vandengriff KD, Winslow RM. New York: Academic Press; 1994: 460-485.
